# Identification of Exhaled Metabolites Correlated with Respiratory Function and Clinical Features in Adult Patients with Cystic Fibrosis by Real-Time Proton Mass Spectrometry

**DOI:** 10.3390/biom14091189

**Published:** 2024-09-21

**Authors:** Malika Mustafina, Artemiy Silantyev, Stanislav Krasovskiy, Alexander Chernyak, Zhanna Naumenko, Aleksandr Suvorov, Daria Gognieva, Magomed Abdullaev, Olga Suvorova, Anna Schmidt, Aida Gadzhiakhmedova, Aleksandra Bykova, Sergey Avdeev, Vladimir Betelin, Abram Syrkin, Philipp Kopylov

**Affiliations:** 1 Department of Cardiology, Functional and Ultrasound Diagnostics, I.M. Sechenov First Moscow State Medical University (Sechenov University), Moscow 119991, Russia; gognieva_d_g@staff.sechenov.ru (D.G.); kopylov_f_yu@staff.sechenov.ru (P.K.); 2 Pulmonology Research Institute under the Federal Medical and Biological Agency of Russia, Moscow 115682, Russia; 3 Research Institute for Systemic Analysis of the Russian Academy of Sciences, Moscow 117218, Russia; abdullaev_m_g@student.sechenov.ru (M.A.);; 4 World-Class Research Center “Digital Biodesign and Personalized Healthcare”, I.M. Sechenov First Moscow State Medical University (Sechenov University), Moscow 119991, Russia; artsilan@gmail.com (A.S.); suvorov_a_yu_1@staff.sechenov.ru (A.S.);; 5 Pulmonology Department, I.M. Sechenov First Moscow State Medical University (Sechenov University), Moscow 119991, Russia

**Keywords:** exhaled biomarkers, volatile organic compounds, cystic fibrosis, proton mass spectrometry, respiratory function

## Abstract

Cystic fibrosis (CF) is a hereditary disease characterized by the progression of respiratory disorders, especially in adult patients. The purpose of the study was to identify volatile organic compounds (VOCs) as predictors of respiratory dysfunction, chronic respiratory infections of *Staphylococcus aureus*, *Pseudomonas aeruginosa*, *Burkholderia cepacia*, and VOCs associated with severe genotype and highly effective modulator treatment (HEMT). Exhaled breath samples from 102 adults with CF were analyzed using PTR-TOF-MS, obtained during a forced expiratory maneuver and normal quiet breathing. Using cross-validation and building gradient boosting classifiers (XGBoost), the importance of VOCs for functional and clinical outcomes was determined. The presence of the previously identified VOCs indole, phenol, and dimethyl sulfide were metabolic outcomes associated with impaired respiratory function. New VOCs associated with respiratory disorders were methyl acetate, carbamic acid, 1,3-Pentadiene, and 2,3-dimethyl-2-butene; VOCs associated with the above mentioned respiratory pathogens were non-differentiable nitrogen-containing organic compounds *m*/*z* = 47.041 (CH5NO)+ and *m*/*z* = 44.044 (C2H5NH+), hydrocarbons (cyclopropane, propene) and methanethiol; and VOCs associated with severe CFTR genotype were non-differentiable VOC *m*/*z* = 281.053. No significant features associated with the use of HEMT were identified. Early non-invasive determination of VOCs as biomarkers of the severity of CF and specific pathogenic respiratory flora could make it possible to prescribe adequate therapy and assess the prognosis of the disease. However, further larger standardized studies are needed for clinical use.

## 1. Introduction

Cystic fibrosis (CF) is a hereditary disease primarily affecting the respiratory system [1]. The disease is characterized by damage to the exocrine glands, as well as vital organs and systems, including the respiratory and gastrointestinal tracts, pancreas, liver, salivary and sweat glands, and the reproductive system [1]. Despite early diagnosis in the form of neonatal screening, early diagnosis of exacerbations of the disease and the composition of the pathogenic flora of the respiratory tract remains important. Persistent chronic respiratory infection reduces the quality of life and the life expectancy of patients with CF [2]. To dynamically assess the patient’s condition, respiratory function studies are used, as follows: spirometry with an assessment of the ratio of forced expiratory volume in the first second (FEV_1_) and forced vital capacity (FVC), and also, less commonly, a study of the diffusion capacity of the lungs for carbon monoxide (DLCO). A decrease in these indicators suggests exacerbation of the disease and a poor prognosis [3]. However, it is necessary to perform the study in the hospital to obtain adequate reproducibility. In addition, not all pediatric patients can perform high-quality spirometry. Early diagnosis of the specific bacterial flora in the respiratory tract is of great importance for adequate therapy. This reduces the risk of antibiotic resistance and improves the prognosis of the disease. These challenges can be addressed through the development of new diagnostic methods aimed at identifying biomarkers for specific pathogenic microbes in the respiratory tract of CF patients. 

Non-invasive diagnostics of the molecular composition of exhaled air (volatilome) as a reflection of pathophysiology and the influence of bacterial flora on the severity of the disease is becoming increasingly important [4]. Determining the microcomposition of exhaled air is one of the most complex analytical tasks. In this regard, few physicochemical methods for the determination of trace amounts of gaseous substances have found their application in this field of research. The most widespread among them are methods for determining volatile organic compounds (VOCs) [5,6,7,8,9,10,11]. 

Previous studies of exhaled breath in CF have analyzed disease-specific VOC profiles [5,6,7], as well as biomarkers specific to *Pseudomonas aeruginosa* [8,9,10,11] or *Staphylococcus aureus* [12,13] and changes in exhaled breath composition during the exacerbation of CF [14,15,16]. However, very few studies have been conducted to analyze the relationship between VOCs and the severity of respiratory disorders and therapy, and all of them involved bag sampling [6,17].

Real-time breath testing could provide representative data with minimal distortion associated with sample collection and storage. One such method is proton transfer reaction time-of-flight mass spectrometry (PTR-TOF-MS). The high ionization efficiency achieved by using PTR-TOF-MS in combination with mass selective detection allows measurements of volatile substances with concentrations of less than 10 pptv. Consumables required during the studies are inexpensive, no additional sample preparation is required, and therefore the analysis of VOCs in exhaled air using PTR-MS is extremely economical compared with other methods of mass spectrometric analysis. 

This study is a continuation of previous work by our team [18]. In our previous study we analyzed the exhaled breath from 102 adult patients with CF compared with a control group using real-time PTR-TOF-MS. Five VOCs that are potentially associated with CF have been identified: indole, phenol, dimethyl sulfide, and two unidentified VOCs with mass-to-charge ratios (*m*/*z*) of 297.0720 and 281.0533. The purpose of this work was to determine VOCs as predictors of severe respiratory disorders, as well as to identify the relationship of VOC spectra with pathogenic respiratory flora and treatment characteristics in patients with CF.

## 2. Materials and Methods

### 2.1. Study Design and Participants

We previously analyzed exhaled breath samples from patients with CF in comparison with controls and published the findings [18]. In the current work, we used data from only 102 patients with CF aged 16 to 62 years, without data from a control group. Spirometry and diffusion tests were performed on Viasys MasterScreen equipment (Viasys Healthcare GMBH, Germany, Höchberg). In addition to clinical and respiratory function data, exhaled air was analyzed using real-time PTR-TOF-MS (Ionicon, Austria, Innsbruck). Based on the examination results and analysis of exhaled air data, VOCs were identified as potential biomarkers for assessing the severity of CF. The PTR-TOF-MS was located in a hospital, allowing all examinations, including the collection of exhaled air samples from CF patients, to be conducted on site. The breath analysis methodology and instrumentation details of PTR-TOF-MS have been described in more detail in our previous study [17]. The study was approved by I.M. Sechenov First Moscow State Medical University ethical committee, Russian Federation, Moscow (Protocol No. 02-23 of 26 January 2023) and registered at ClinicalTrials.gov (NCT05727852). Written informed consent was obtained from all study participants.

### 2.2. PTR-TOF Data Preprocessing

Preprocessing of the raw data was performed using an original algorithm implemented in Python (3.9) using the h5py (3.9.0), scipy (1.11.1), and pandas (2.0.3) packages. For each sample and each spectrum in the sample, recalibration was carried out using the following three ions of the Ionicon Permeation Source for Calibration (PerMaSCal) system: 21.0220, 203.94299, 330.85 *m*/*z*.

Capnostat readings were used for additional quality control. If the capnostat signal for a sample exceeded the cutoff of 3.5 units, then such a sample was excluded from further processing. For a more accurate assessment of the patient’s inhalation-exhalation, capnostat readings were not used as since they had a noticeable inertia relative to the water adduct signal with the hydronium ion. For each spectrum in the sample, the ion current (EIC) was extracted for three biogenic ions ([M + H]^+^) with *m*/*z* = 69.07 (isoprene), *m*/*z* = 63.02 (dimethyl sulfide), and *m*/*z* = 55.03 (1,2-butadiene), as well as capnostat readings and an ion with a mass of *m*/*z* = 37.038 corresponding to the adduct for water with the hydronium ion [H_2_O + H_3_O]^+^. The response of the water-hydronium ion adduct correlated with the amount of exhaled moisture and was used to detect the patient’s inhalation–exhalation cycles during breathing. Spectra were collected for samples where the water adduct signal exceeded the cutoff (2 × 10^5^ cps) and showed a local maximum corresponding to 2 of the 3 biogenic ions. We also used several additional criteria for quality control of the samples received for processing. We checked the mass error for calibrant ions released by the PerMaSCal system at 21.022, 203.94, and 330.85 *m*/*z*. The permissible mass error level for each calibrant had to be less than 100 ppm, with typical errors ranging from 20 to 30 ppm. Additionally, we estimated the number of local maxima corresponding to exhalation cycles. Samples characterized by breathing with fewer than 3 inhalation–exhalation cycles, were excluded from data processing.

The selected spectra within each sample are summed up, averaged, and smoothed by the Savitzky–Golay filter. For the resulting averaged process, ion peaks are selected and filtered. Selected ions are aligned between samples if they occur in more than 50% of the samples examined. The compiled library of mass-to-charge ratio relations is used to extract signal areas for ions in a ±0.015–0.4 *m*/*z* window for selected spectra by equation 2 × *m*/*z* × √(*m*/*z*)/√(reference_*m*/*z*)/resolution. The data for each patient is averaged, normalized to the signal of the cluster heavy water isotope with hydroxonium ion ([D_2_O + H_3_O]^+^), and then used for further statistical processing.

### 2.3. Formula Annotation

For significantly different features, annotation was performed based on the detected ion mass-to-charge ratio. The search database was created from two proprietary Ionicon ion databases (300 and 1000 factory masses) and chemical compounds described in the literature as a CF marker with a maximum permissible error of ±200 ppm (Table S1). The annotation results are shown in Table S2.

### 2.4. Statistical Analysis

#### 2.4.1. Descriptive Statistics

For quantitative indicators, the nature of the distribution (using the Shapiro-Wilk test), the average value, standard deviation, median, interquartile, 95% confidence interval, and minimum and maximum values were determined. For categorical and qualitative features, the proportion and the absolute number of values were determined. 

#### 2.4.2. Role of VOCs as Predictors of Respiratory Dysfunction

The VOCs, identified from our previous study [18], were assessed using three endpoints—a decrease below the threshold of −1.645 in normalized respiratory parameters FEV_1_/FVC z-scores, FEV_75_ z-scores, and DLCO z-scores [19]—analyzed separately for forced and normal exhalation. The process involved repeated cross-validation, data normalization, and building gradient boosting classifiers (XGBoost) on each split to assess feature importances and overall model quality using the area under the receiver operating characteristic curve (AUC).

#### 2.4.3. Selection of VOCs and Relation to the Endpoints

To select predictors, cross-validation was carried out, during which the data were transformed and the classifier was fitted to assess the importance of predictors in a single model. Potential predictors included only VOCs, calibrant molecules were removed from the data, as well as substances with a *m*/*z* less than 42.

#### 2.4.4. Feature Selection

Due to the small number of observations, random sampling of 2/3 of the full dataset was performed 1000 times. In each repetition, data preprocessing was carried out which involved normalization and iterative imputation using Bayesian ridge regression for quantitative data. There were no categorical or binary features. During each repetition, an XGBoost classifier was fitted to calculate feature importance. Feature importances for every VOC over the previous 1000 iterations were then averaged using the median calculation and sorted from the highest median to the lowest values. The feature selection process was conducted separately for forced and normal breathing. For each endpoint, the top 30 VOCs with the highest median scores from both forced expiratory maneuver and normal quiet breathing were selected to identify intersections. VOCs common to both types of breathing were used for further analysis.

#### 2.4.5. Assessment of the Relationship of Selected Characteristics with the Outcome

For each endpoint, common VOCs were used to fit new XGBoost classifiers (separately for forced and normal expiratory maneuvers) on the full dataset using leave-one-out cross-validation. The probabilities of the presence of each outcome were calculated to determine the quality of the constructed classifiers, as well as sensitivity and specificity, positive and negative predictive value based on a probability threshold of 0.5. Additionally, for the best models, as a result of cross-validation, feature importances were redefined to include only common VOCs in the models. Thus, among the common factors, it became possible to identify those whose feature importances did not tend to 0, and also to compare the feature importances for forced and normal expiratory maneuver using the same VOCs.

## 3. Results

### 3.1. Participant Characteristics

A brief description of the patients is presented in Table 1. Detailed data, including mutations, are presented in our previous article [18]. Here we highlight respiratory function scores below the lower limit of normal, which was defined as below a z-score of −1.645, according to the guidelines [19]. All patients were non-smokers. The majority of patients with CF had obstructive disorders, including small airway obstruction, as follows: FEV_1_/FVC z-score less than −1.645 in 81 patients with CF (79.4%) and forced expiratory flow when 75% of FVC has been exhaled (FEF_75_) z-score less than −1.645 in 84 patients with CF (82.4%). Thirty-eight patients (39%) had impaired gas exchange function of the lungs (DLCO z-score less than −1.645). Most patients had a more severe genotype—78 (76.5%)—and only 25 (24.5%) had highly effective modulator treatment (HEMT). Additional demographic characteristics based on respiratory function indicators are presented in Table S3.

### 3.2. Breath Analysis

#### 3.2.1. Predictors of Respiratory Dysfunction

In our previous work, we identified diagnostically significant VOCs for CF, as follows: *m*/*z* = 118.0660 (indole), *m*/*z* = 95.0601 (phenol), *m*/*z* = 63.0165 (dimethyl sulfide), *m*/*z* = 281.0534 ([C_19_H_7_NO_2_, C_12_H_11_NO_7_ and C_16_H_9_O_5_] H^+^), and *m*/*z* = 297.0720 ([C_12_H_13_N_2_O_7_ and C_17_H_13_O_5_] H^+^) [18]. 

The subsequent objective was to determine whether these VOCs were predictors of respiratory dysfunction, which was defined as a decrease in the FEV_1_/FVC, FEF_75_ and DLCO z-score of less than −1.645 using XGBoost (Table 2). 

Table 2 demonstrates that almost all of the indicated VOCs contribute significantly to the development of respiratory disorders (obstruction and impaired gas exchange function of the lungs). The composite model for the endpoints of FEV_1_/FVC, FEF_75_ and DLCO with z-scores of less than −1.645 in the **forced expiratory maneuver** included *m*/*z* = 118.0660 (indole), *m*/*z* = 95.0601 (phenol), *m*/*z* = 63.0165 (dimethyl sulfide), *m*/*z* = 281.0534 ([C_19_H_7_NO_2_, C_12_H_11_NO_7_ and C_16_H_9_O_5_] H^+^), and *m*/*z* = 297.0720 ([C12H13N2O7 and C17H13O5] H+) and had respective AUCs of 0.93 (95% CI: 0.86–0.98), 0.92 (95% CI: 0.82–0.98) and 0.96 (95% CI: 0.93–0.99) during cross-validation. The composite model for endpoints of FEV_1_/FVC, FEF_75_ and DLCO with a z-score of less than −1.645 in **normal quiet breathing** included the same VOCs and had respective AUCs of 0.96 (95% CI: 0.92–0.99), 0.93 (95% CI: 0.94–0.99) and 0.84 (95% CI: 0.75–0.91). 

#### 3.2.2. Selection of New VOCs Significant for Functional and Clinical Endpoints

In addition to the five previously obtained biomarkers, the aim of our work was to identify VOCs specific to a variety of endpoints, associated with the severe course of CF. Eight outcomes included the “severity” of mutation of the CF transmembrane conductance regulator gene (CFTR, classes 1, 2 or 3 [20]; the use of highly effective modulator treatment (HEMT); bacterial colonization of *Staphylococcus aureus*, *Pseudomonas aeruginosa*, and *Burkholderia cepacia* (determined by identifying the pathogen in at least two consecutive sputum cultures); and a decrease below the threshold of −1.645 for the following normalized respiratory parameters: FEV_1_/FVC z-scores, FEV_75_ z-scores, and DLCO z-scores. After selecting features based on XGBoost and calculating medians for feature importances, intersections by VOCs for each outcome were determined (Table S2).

Common VOCs for normal and forced exhalation were included in further analysis to establish their relationships with the outcomes. 

The classifiers were fitted using only these VOCs for each breathing type. Cross-validation using the leave-one-out method was used to select the best XGBoost hyperparameters. For the best estimator, feature importances for each outcome were assessed in accordance with different types of breathing, and probability predictions were calculated to estimate the quality of the built classifiers, which reflected the quality of the relationship between VOCs and the outcomes. The composite model for functional and clinical endpoints during both the forced expiratory maneuver and normal quiet breathing included selected VOCs, which are presented for each endpoint in Table S2. The results for AUC, sensitivity and specificity are presented in Table 3.

The selection of significant predictors of functional and clinical outcomes using the XGBoost algorithm is presented in Table 4.

**Figure 1:**
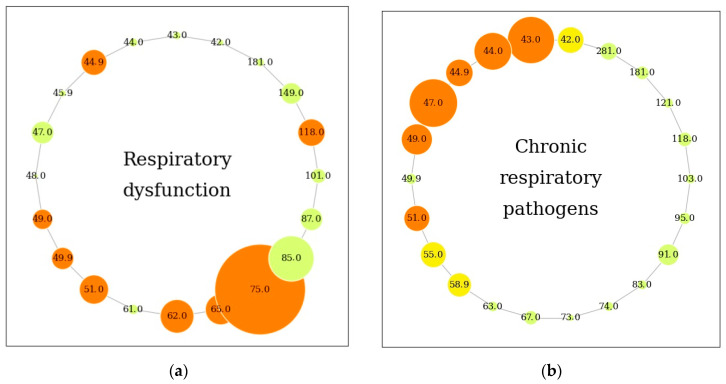

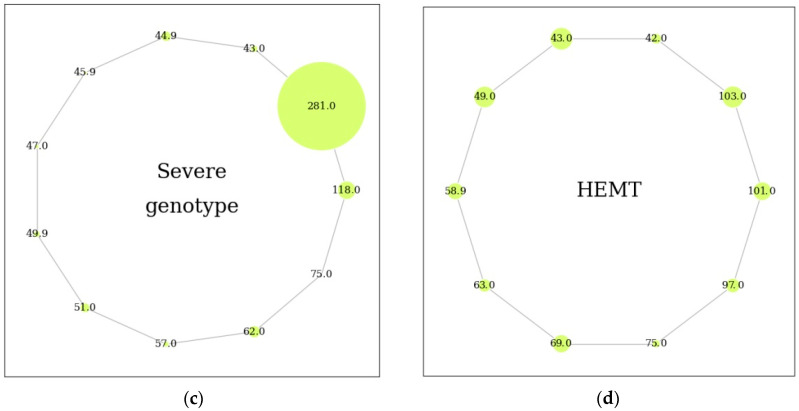
Features for respiratory and clinical outcomes in CF. (**a**) Respiratory dysfunction—obstructive disorders (FEV_1_/FVC < LLN) and gas exchange dysfunction (DLCO < LLN). (**b**) Chronic respiratory pathogens: *S. aureus*, *P. aeruginosa*, *B. cepacia*—determination of the pathogen during bacteriological examination of sputum at least 2 times in a row. (**c**) Severe genotype of cystic fibrosis transmembrane conductance regulator gene (CFTR)—class 1, 2, or 3 of CFTR mutation [20]. (**d**) HEMT—highly effective modulator treatment. DLCO: diffusing capacity of the lungs for carbon monoxide; FEV_1_: forced expiratory volume in 1 s; FVC: forced vital capacity; LLN: lower limit of normal (z-score < −1.645). The size of the node is determined by the total importance of the feature, both during a forced and normal expiratory maneuver (the higher the importance of the feature, the larger the size of the node). The color of a node is determined by the frequency of occurrence of the feature in different outcomes. Bright orange nodes reflect the frequency of the feature in more than 2 outcomes. The number in the node indicates the mass-to-charge ratio (*m*/*z*) of the volatile organic compound (VOC).

The importance of features was summarized for both forced and normal expiratory maneuvers and is presented graphically for respiratory disorders, bacterial pathogens (*S. aureus*, *P. aeruginosa*, *B. cepacia*), for the “severe” genotype of the gene CFTR and for HEMT (Figure 1).

Thus, the most significant predictors for respiratory disorders were found to be *m*/*z* = 75.042 (methyl acetate), *m*/*z* = 62.028 (methyl nitrite), *m*/*z* = 65.057 (2-fluoroethanol), *m*/*z* = 118.066 (indole) and, especially for gas exchange disorders, *m*/*z* = 85.077 (2,3-dimethyl-2-butene); for respiratory pathogens in general, we found the following: non-differentiable nitrogen-containing organic compounds *m*/*z* = 47.041 (CH_5_NO)^+^ and *m*/*z* = 44.044 (C_2_H_5_NH^+^), hydrocarbons (cyclopropane, propene) *m*/*z* = 43.046 (C_3_H_6_H^+^), sulfur compound *m*/*z* = 49.020 (methanethiol); and, for the severe CFTR genotype, the most significant was predictor was found to be non-differentiable compound *m*/*z* = 281.053. No significant features associated with the use of HEMT were identified.

## 4. Discussion

This study is the first to use PTR-TOF-MS in a large sample of adult patients with CF to analyze exhaled biomarkers of respiratory disorders and clinical features of the disease. 

In the first part of this study, we determined that previously identified biomarkers of CF, indole, phenol, dimethyl sulfide, *m*/*z* = 281.053, and *m*/*z* = 297.072 [18] are also predictors of impaired respiratory function in patients with this disease. As we previously suggested, these markers may indicate dysbiosis of the intestinal microflora and impaired liver function in patients with CF. It can be assumed that, in adult patients with CF, who, as a rule, have a progression of respiratory disorders over time, the phenomena of intestinal dysbiosis will also worsen due to long-term antibiotic therapy, which is associated with indole, phenol, and dimethyl sulfide.

In the second part of our study, we identified new VOCs as predictors of obstructive and gas exchange disorders in CF. Indole was again included in this group of biomarkers as a predictor of obstructive disorders, including obstruction of small airways. The most significant predictor of obstructive disorders was methyl acetate. Methyl acetate is an ester formed as a result of the metabolism of acetic acid and methanol. An earlier study showed an increase in acetic acid concentrations in patients with CF [10]. Methanol in exhaled air was also important in distinguishing patients with CF from the control group [21] and as a marker of exacerbation of the disease [14]. Methyl acetate has been shown to be produced by bronchial epithelial cells in vitro [22]. This metabolite itself was one of the markers of CF in exhaled breath analysis, which could be a sign of neutrophilic inflammation in these patients [23]. It has been suggested that carbamic acid is one of the metabolites produced by *Escherichia coli*. Martinez-Medina et al. found that dysbiosis in CF leads to increased colonization of the intestinal mucosa by *E. coli,* resulting in inflammation [24]. Chronic intestinal inflammation, in turn, can lead to impaired lipid absorption and aggravate the overall progression of the disease.

Previous studies of exhaled breath and respiratory function in patients with CF have primarily assessed FEV_1_ [6,25]. Our study is the first to identify a predictor of impaired gas exchange function in patients with CF—hydrocarbon, 2,3-dimethyl-2-butene. Methylated hydrocarbons are thought to result from lipid peroxidation [26]. Chronic hypoxia, which is manifested by a gas exchange dysfunction, triggers lipid peroxidation reactions, the marker of which can be identified by 2,3-dimethyl-2-butene. In a study by Papaefstathiou et al., 2,3-dimethyl-2-butene was associated with smoking, confirming the non-specificity of this marker [27]. 

Volatile nitrogen-containing (VNCs) and volatile sulfur-containing organic compounds (VSCs) were identified in our study as predictors of chronic respiratory infection in general. This is consistent with early studies where hydrogen cyanide and 2′-aminoacetophenone in exhaled breath were associated with *P. aeruginosa* in patients with CF [9,28,29]. This infectious agent feeds on sulfate, which leads to the formation of VSCs such as carbon disulfide and dimethyl sulfide, as shown by in vitro and in vivo studies [5,18,30]. If we analyze predictors for a single infectious pathogen in our study, a VSC, dimethyl sulfide, and an aromatic heterocycle, indole, were also found to be the most important predictor for *P. aeruginosa*. Another aromatic heterocycle, phenol, as a possible result of intestinal dysbiosis in adult patients with CF due to long-term antibiotic therapy, was significant in our study as a predictor of *S. aureus*, identified both during forced and normal expiratory maneuvers. Lactic acid was an important predictor for *S. aureus,* especially when analyzing forced expiratory metabolites. This metabolite is formed as a result of chronic hypoxia and ketoacidosis, which probably occurs in patients with CF. It has been previously described that exhaled lactic acid concentrations are increased in patients with CF during exacerbation [15]. 

For the first time in our study, we examined possible predictors of one of the most severe bacterial infections in CF, *B. cepacia*. One significant predictor was acetonitrile, which has also been previously described in exhaled breath analysis of patients with CF compared with controls in a study by Gramacho et al. [31]. Acetonitrile can be synthesized from hydrogen cyanide, which is described above as an important marker of *P. aeruginosa*, by replacing the hydrogen with a methyl group. *B. cepacia* and *P. aeruginosa* are known to interact and can form mixed biofilms in the lung tissue of patients with CF [32]. This can also be manifested by the biochemical transformations of the metabolites of the vital activity of both of these microorganisms. Acetonitrile is also observed in significant quantities in the breath of smokers [33], though all patients with CF in our study were non-smokers. Methylidynephosphane, another *B. cepacia* predictor, can also be formed from hydrogen cyanide when nitrile nitrogen is replaced by phosphorus. Aromatic hydrocarbons, 1,3-dimethylbenzene or 1,4-dimethylbenzene (m-/p-xylene), which were found to be significant as a marker of *B. cepacia* in our research, have also been described in a study by Horck et al. when studying the composition of exhaled air during exacerbation of CF in children [16].

For the first time, we attempted to analyze the association of severe variants of the CFTR gene mutation with exhaled air metabolites. The most significant was the undifferentiated VOC *m*/*z* = 281.053, which also turned out to be another important predictor of *B. cepacia*. In addition, VOCs associated with the therapeutic intervention in our study were analyzed. Previous studies have been conducted to analyze VOCs in relation to hormonal and antibacterial therapy in patients with CF [34]. Woollam et al. also described increased aldehyde concentrations in patients not receiving HEMT [17]. As can be seen in Figure 1d, our study failed to identify the most significant VOC in HEMT. However, we can still assume that 1,4-dimethylpyrazole, methanethiol, and isopropyl acetate are, to some extent, associated with HEMT intake. These results may be due to the limited number of patients receiving HEMT, which included only 25 adults with CF from the total sample.

The analytical method PTR-TOF-MS is characterized by a fairly accurate determination of the mass of VOCs. However, challenges arise in the annotation of exhaled metabolites due to the inability to separate isobaric compounds and the coincidence of ion peaks. As a result, some VOCs cannot be annotated using the IONICON databases or the existing literature on VOCs in CF and are instead presented in Table 4 as exact masses. Another limitation of our study was the relatively small number of patients. Due to the small sample, feature selection and validation were performed using different types of cross-validation, mainly to increase dispersion. This approach significantly overestimates the quality scores of the models and cannot safeguard against various biases. Consequently, the generalizability of the research findings is uncertain, and the results should be considered hypothesis generating. A much larger sample is needed for further confirmatory studies. In addition, expiratory sampling was not standardized by flow and capnography, although the forced expiratory maneuver and normal quiet breathing were analyzed separately. The absence of such standardization limits the ability to differentiate between the contributions of the upper and lower respiratory tracts to the samples and hinders precise analysis of the alveolar portion of the exhalation.

## 5. Conclusions

In conclusion, VOCs determined by PTR-TOF-MS could serve as significant biomarkers of severe respiratory disorders and of specific bacterial agents such as *S. aureus*, *P. aeruginosa*, and *B. cepacia* in patients with CF. In addition, identifying VOCs associated with a more severe genotype of CF could enable the prediction of disease progression and support the timely prescription of appropriate therapy. Further large-scale standardized studies are required to validate previously identified biomarkers and potentially discover new ones.

## Figures and Tables

**Table 1:** Participant characteristics.

Patients [N]	102
Age, years [Mean (SD)]	25.6 ± 7.8
Gender—Males [N (%)]	54 (52.9%)
BMI kg·m^2^ [Mean (SD)]	19.8 ± 3.6
mMRC, score	1.1 ± 0.8
FVC < LLN [N (%)]	58 (56.9%)
FEV_1_ < LLN [N (%)]	84 (82.4%)
FEV_1_/FVC < LLN [N (%)]	81 (79.4%)
FEF_75_ < LLN [N (%)]	84 (82.4%)
DLCO < LLN [N (%)]	39 (38.2%)
Age of diagnosis, years [Mean (SD)]	6.8 ± 9.9
Mutation, mild/severe [N (%)]	24 (23.5%)/78 (76.5%)
Culture positive [N (%)]	
*Staphylococcus aureus*	40 (39.2%)
*Pseudomonas aeruginosa*	57 (55.9%)
*Burkholderia cepacia*	5 (4.9%)
HEMT [N (%)]	25 (24.5%)

BMI: Body mass index, DLCO: diffusing capacity of the lungs for carbon monoxide, FEV_1_: forced expiratory volume in 1 s, FVC: forced vital capacity, FEF_75_: the forced expiratory flow when 75% of FVC has been exhaled, HEMT: highly effective modulator treatment; mMRC: Modified Medical Research Council, LLN: lower limit of normal, and SD: standard deviation.

**Table 2:** Predictors of the respiratory dysfunction by the XGBoost algorithm.

Forced Expiratory Maneuver			
Predictors	FEV_1_/FVC, feat.imp. *	FEF_75_, feat.imp. *	DLCO, feat.imp. *
*m*/*z* = 63.0165 (dimethyl sulfide)	0.130	0.224	0.293
*m*/*z* = 95.0601 (phenol)	0.130	0.079	0.153
*m*/*z* = 118.0660 (indole)	0.316	0.139	0.301
*m*/*z* = 281.0534 ([C_19_H_7_NO_2_, C_12_H_11_NO_7_ and C_16_H_9_O_5_] H^+^)	0.321	0.128	0.139
*m*/*z* = 297.0720 ([C_12_H_13_N_2_O_7_ and C_17_H_13_O_5_] H^+^)	0.103	0.431	0.114
Normal Quiet Breathing			
Predictors	FEV_1_/FVC, feat.imp.	FEF_75_, feat.imp.	DLCO, feat.imp.
*m*/*z* = 63.0165 (dimethyl sulfide)	0.209	0.237	0.053
*m*/*z* = 95.0601 (phenol)	0.132	0.025	0.222
*m*/*z* = 118.0660 (indole)	0.269	0.392	0.006
*m*/*z* = 281.0534 ([C_19_H_7_NO_2_, C_12_H_11_NO_7_ and C_16_H_9_O_5_] H^+^)	0.200	0.122	0.089
*m*/*z* = 297.0720 ([C_12_H_13_N_2_O_7_ and C_17_H_13_O_5_] H^+^)	0.189	0.224	0.631

DLCO: diffusing capacity of the lungs for carbon monoxide, FEV_1_: forced expiratory volume in 1 s, FVC: forced vital capacity, FEF_75_: forced expiratory flow when 75% of FVC has been exhaled. * Endpoints (z-score < −1.645), feat.imp.—feature importances according to XGBoost.

**Table 3:** Results of the composite model for clinical and functional outcomes based on selected VOCs during normal and forced exhale.

Clinical and Functional Endpoints	Forced Expiratory Maneuver				Normal Quiet Breathing			
	AUC	95% CI	Sensitivity	Specificity	AUC	95% CI	Sensitivity	Specificity
FEV_1_/FVC < LLN	0.980	0.960–1.000	0.947	0.952	0.968	0.938–0.999	0.947	0.905
FEF_75_ < LLN	0.994	0.984–1.000	0.980	0.944	0.989	0.971–1.000	0.959	1.000
DLCO < LLN	0.999	0.996–1.000	0.974	0.974	0.994	0.985–1.000	0.949	0.974
*P. aeruginosa*	1.000	0.988–1.000	1.000	1.000	0.997	0.990–1.000	1.000	1.000
*S.* *aureus*	1.000	0.960–1.000	1.000	1.000	0.999	0.997–1.000	1.000	1.000
*B.* *cepacia*	0.996	0.988–1.000	1.000	1.000	0.993	0.977–1.000	1.000	1.000
“Severe” genotype *	0.981	0.960–1.000	0.888	0.958	0.969	0.939–1.000	0.933	0.917
HEMT	1.000	0.988–1.000	0.960	1.000	0.981	0.960–1.000	0.880	0.956

AUC: area under the receiver operating characteristic curve; DLCO: diffusing capacity of the lungs for carbon monoxide; FEV_1_: forced expiratory volume in 1 s; FVC: forced vital capacity; FEF_75_: forced expiratory flow when 75% of FVC has been exhaled; HEMT: highly effective modulator treatment; LLN: lower limit of normal (z-score < −1.645); * Class 1, 2 or 3 of CFTR mutation [20].

**Table 4:** Predictors of functional and clinical outcomes by the XGBoost algorithm.

Clinical and Functional Endpoints	Forced Expiratory Maneuver			Normal Quiet Breathing		
	*m*/*z*	VOC Predictors	Feature Importances	*m*/*z*	VOC Predictors	Feature Importances
FEV_1_/FVC < LLN	75.042	Methyl acetate	0.306	75.042	Methyl acetate	0.425
	44.992	Methinophosphide	0.165	62.028	Carbamic acid	0.119
	118.066	Indole	0.163	51.037	CH_6_O_2_H^+^	0.099
	51.037	CH_6_O_2_H^+^	0.120	44.992	Methinophosphide	0.083
	62.028	Carbamic acid	0.086	181.007	p-Xylene	0.081
	61.033	Acetic acid	0.072	118.066	Indole	0.077
	44.044	C2H5NH^+^	0.059	61.033	Acetic acid	0.061
	181.007	p-Xylene	0.028	44.044	C_2_H_5_NH^+^	0.054
FEF_75_ < LLN	47.039	(CH_5_NO)^+^	0.252	75.046	Methyl acetate	0.283
	75.042	Methyl acetate	0.168	62.028	Carbamic acid	0.101
	62.028	Carbamic acid	0.133	65.057	1,3-Pentadiene	0.095
	49.020	Methanethiol	0.096	51.038	CH_6_O_2_H^+^	0.074
	51.037	CH_6_O_2_H^+^	0.088	45.992	NO_2_^+^	0.066
	49.994	H_2_O_3_^+^	0.070	118.070	Indole	0.066
	118.066	Indole	0.050	43.047	C_3_H_6_H^+^	0.061
	65.046	1,3-Pentadiene	0.046	49.994	H_2_O_3_^+^	0.061
	43.046	C_3_H_6_H^+^	0.043	44.991	Methinophosphide	0.056
	44.992	Methinophosphide	0.032	48.045	Methoxyamine	0.055
	45.994	NO_2_^+^	0.023	47.041	(CH_5_NO)^+^	0.043
	48.044	Methoxyamine	0.000	49.004	Methanethiol	0.039
DLCO < LLN	85.077	2,3-dimethyl-2-butene	0.283	85.095	2,3-dimethyl-2-butene	0.310
	149.096	3-nitrobenzonitrile	0.230	65.057	1,3-Pentadiene	0.141
	87.065	2-Pentanone (3-methyl-2-butanone)	0.162	87.078	2-Pentanone (3-methyl-2-butanone)	0.132
	65.046	1,3-Pentadiene	0.119	101.043	C5H8O2H^+^	0.126
	101.050	C_5_H_8_O_2_H^+^	0.066	49.994	H_2_O_3_^+^	0.098
	49.994	H_2_O_3_^+^	0.060	49.004	Methanethiol	0.080
	49.020	Methanethiol	0.048	149.099	3-nitrobenzonitrile	0.050
	42.030	Acetonitrile	0.032	42.032	Acetonitrile	0.047
*S. aureus*	91.053	Lactic acid	0.222	47.041	(CH_5_NO)^+^	0.147
	55.039	C_2_H_2_N_2_H^+^	0.134	58.960	NA	0.108
	67.055	1,3-Cyclopentadiene	0.109	51.038	CH_6_O_2_H^+^	0.106
	121.087	4-Methylbenzaldehyde	0.095	95.054	Phenol	0.083
	95.060	Phenol	0.076	67.056	1,3-Cyclopentadiene	0.079
	49.020	Methanethiol	0.060	43.047	C_3_H_6_H^+^	0.076
	83.085	Cyclopropane, (1-methylethenyl)-	0.058	49.004	Methanethiol	0.074
	47.039	(CH_5_NO)^+^	0.057	83.086	Cyclopropane, (1-methylethenyl)-	0.070
	51.037	CH_6_O_2_H^+^	0.052	91.055	Lactic acid	0.063
	43.046	C_3_H6H^+^	0.046	44.048	C_2_H_5_NH^+^	0.053
	44.992	Methinophosphide	0.038	44.991	Methinophosphide	0.053
	58.952	NA	0.029	55.039	C_2_H_2_N_2_H^+^	0.049
	44.044	C_2_H_5_NH^+^	0.024	121.094	4-Methylbenzaldehyde	0.037
*P. aeruginosa*	47.039	(CH_5_NO)^+^	0.145	44.048	C_2_H_5_NH^+^	0.115
	44.044	C_2_H_5_NH^+^	0.101	58.960	NA	0.106
	118.066	Indole	0.095	47.041	(CH_5_NO)^+^	0.098
	74.058	Acetamide, N-methyl-	0.086	118.070	Indole	0.094
	58.952	NA	0.077	49.994	H_2_O_3_^+^	0.088
	43.046	C_3_H_6_H^+^	0.075	63.017	Dimethyl sulfide	0.079
	103.072	Isopropyl acetate	0.074	44.991	Methinophosphide	0.071
	44.992	Methinophosphide	0.073	42.032	Acetonitrile	0.068
	49.994	H_2_O_3_^+^	0.057	43.047	C_3_H_6_H^+^	0.056
	63.016	Dimethyl sulfide	0.057	103.068	Isopropyl acetate	0.054
	49.020	Methanethiol	0.054	73.065	Methyl ethyl ketone, 2-buten-1-ol	0.053
	73.063	Methyl ethyl ketone, 2-buten-1-ol	0.037	49.004	Methanethiol	0.049
	42.030	Acetonitrile	0.036	74.061	Acetamide, N-methyl-	0.041
	51.037	CH_6_O_2_H^+^	0.032	51.038	CH_6_O_2_H^+^	0.029
*B. cepacia*	43.046	C_3_H_6_H^+^	0.168	43.047	C_3_H_6_H^+^	0.201
	44.044	C_2_H_5_NH^+^	0.151	42.032	Acetonitrile	0.171
	181.007	p-Xylene	0.122	281.066	NA	0.134
	49.020	Methanethiol	0.110	47.041	(CH_5_NO)^+^	0.097
	281.053	NA	0.099	44.991	Methinophosphide	0.088
	47.039	(CH_5_NO)^+^	0.095	55.039	C_2_H_2_N_2_H^+^	0.088
	42.030	Acetonitrile	0.076	49.004	Methanethiol	0.062
	55.039	C_2_H_2_N_2_H^+^	0.072	181.014	p-Xylene	0.056
	51.037	CH_6_O_2_H^+^	0.071	44.048	C_2_H_5_NH^+^	0.052
	44.992	Methinophosphide	0.037	51.038	CH_6_O_2_H^+^	0.051
“Severe” genotype *	281.053	NA	0.545	281.066	NA	0.517
	118.066	Indole	0.097	118.070	Indole	0.122
	62.028	Carbamic acid	0.061	62.028	Carbamic acid	0.084
	57.065	1-Propene, 2-methyl-	0.059	51.038	CH_6_O_2_H^+^	0.074
	44.992	Methinophosphide	0.055	44.991	Methinophosphide	0.067
	51.037	CH_6_O_2_H^+^	0.041	43.047	C_3_H_6_H^+^	0.060
	45.994	NO_2_^+^	0.040	49.994	H_2_O_3_^+^	0.043
	49.994	H_2_O_3_^+^	0.035	47.041	(CH_5_NO)^+^	0.022
	47.039	(CH_5_NO)^+^	0.028	57.069	1-Propene, 2-methyl-	0.010
	43.046	C_3_H_6_H^+^	0.023	75.046	Methyl acetate	0.001
	75.042	Methyl acetate	0.016	45.992	NO_2_^+^	0.000
HEMT	101.050	C_5_H_8_O_2_H^+^	0.170	43.047	C_3_H_6_H^+^	0.208
	97.086	1,4-Dimethylpyrazole	0.131	103.068	Isopropyl acetate	0.152
	49.020	Methanethiol	0.119	49.004	Methanethiol	0.141
	103.072	Isopropyl acetate	0.113	58.960	NA	0.133
	69.071	1,4-Pentadiene (isoprene)	0.110	69.072	1,4-Pentadiene (isoprene)	0.108
	63.016	Dimethyl sulfide	0.097	63.017	Dimethyl sulfide	0.061
	42.030	Acetonitrile	0.085	75.046	Methyl acetate	0.057
	58.952	NA	0.070	101.043	C_5_H_8_O_2_H^+^	0.054
	43.046	C_3_H_6_H^+^	0.065	97.094	1,4-Dimethylpyrazole	0.044
	75.042	Methyl acetate	0.039	42.032	Acetonitrile	0.042
**Clinical and Functional Endpoints**	**Forced Expiratory Maneuver**			**Normal Quiet Breathing**		
	***m*** **/*z***	**VOCs Predictors**	**Feature Importances**	***m*** **/*z***	**VOCs Predictors**	**Feature Importances**
FEV_1_/FVC < LLN	75.042	Methyl acetate	0.306	75.042	Methyl acetate	0.425
	44.992	Methinophosphide	0.165	62.028	Carbamic acid	0.119
	118.066	Indole	0.163	51.037	CH_6_O_2_H^+^	0.099
	51.037	CH_6_O_2_H^+^	0.120	44.992	Methinophosphide	0.083
	62.028	Carbamic acid	0.086	181.007	p-Xylene	0.081
	61.033	Acetic Acid	0.072	118.066	Indole	0.077
	44.044	C2H5NH^+^	0.059	61.033	Acetic Acid	0.061
	181.007	p-Xylene	0.028	44.044	C_2_H_5_NH^+^	0.054
FEF_75_ < LLN	47.039	(CH_5_NO)^+^	0.252	75.046	Methyl acetate	0.283
	75.042	Methyl acetate	0.168	62.028	Carbamic acid	0.101
	62.028	Carbamic acid	0.133	65.057	1,3-Pentadiene	0.095
	49.020	Methanethiol	0.096	51.038	CH_6_O_2_H^+^	0.074
	51.037	CH_6_O_2_H^+^	0.088	45.992	NO_2_^+^	0.066
	49.994	H_2_O_3_^+^	0.070	118.070	Indole	0.066
	118.066	Indole	0.050	43.047	C_3_H_6_H^+^	0.061
	65.046	1,3-Pentadiene	0.046	49.994	H_2_O_3_^+^	0.061
	43.046	C_3_H_6_H^+^	0.043	44.991	Methinophosphide	0.056
	44.992	Methinophosphide	0.032	48.045	Methoxyamine	0.055
	45.994	NO_2_^+^	0.023	47.041	(CH_5_NO)^+^	0.043
	48.044	Methoxyamine	0.000	49.004	Methanethiol	0.039
DLCO < LLN	85.077	2,3-dimethyl-2-butene	0.283	85.095	2,3-dimethyl-2-butene	0.310
	149.096	3-nitrobenzonitrile	0.230	65.057	1,3-Pentadiene	0.141
	87.065	2-Pentanone (3-methyl-2-butanone)	0.162	87.078	2-Pentanone (3-methyl-2-butanone)	0.132
	65.046	1,3-Pentadiene	0.119	101.043	C5H8O2H^+^	0.126
	101.050	C_5_H_8_O_2_H^+^	0.066	49.994	H_2_O_3_^+^	0.098
	49.994	H_2_O_3_^+^	0.060	49.004	Methanethiol	0.080
	49.020	Methanethiol	0.048	149.099	3-nitrobenzonitrile	0.050
	42.030	Acetonitrile	0.032	42.032	Acetonitrile	0.047
*S. aureus*	91.053	Lactic acid	0.222	47.041	(CH_5_NO)^+^	0.147
	55.039	C_2_H_2_N_2_H^+^	0.134	58.960	NA	0.108
	67.055	1,3-Cyclopentadiene	0.109	51.038	CH_6_O_2_H^+^	0.106
	121.087	4-Methylbenzaldehyde	0.095	95.054	Phenol	0.083
	95.060	Phenol	0.076	67.056	1,3-Cyclopentadiene	0.079
	49.020	Methanethiol	0.060	43.047	C_3_H_6_H^+^	0.076
	83.085	Cyclopropane, (1-methylethenyl)-	0.058	49.004	Methanethiol	0.074
	47.039	(CH_5_NO)^+^	0.057	83.086	Cyclopropane, (1-methylethenyl)-	0.070
	51.037	CH_6_O_2_H^+^	0.052	91.055	Lactic acid	0.063
	43.046	C_3_H6H^+^	0.046	44.048	C_2_H_5_NH^+^	0.053
	44.992	Methinophosphide	0.038	44.991	Methinophosphide	0.053
	58.952	NA	0.029	55.039	C_2_H_2_N_2_H^+^	0.049
	44.044	C_2_H_5_NH^+^	0.024	121.094	4-Methylbenzaldehyde	0.037
*P. aeruginosa*	47.039	(CH_5_NO)^+^	0.145	44.048	C_2_H_5_NH^+^	0.115
	44.044	C_2_H_5_NH^+^	0.101	58.960	NA	0.106
	118.066	Indole	0.095	47.041	(CH_5_NO)^+^	0.098
	74.058	Acetamide, N-methyl-	0.086	118.070	Indole	0.094
	58.952	NA	0.077	49.994	H_2_O_3_^+^	0.088
	43.046	C_3_H_6_H^+^	0.075	63.017	Dimethyl sulfide	0.079
	103.072	Isopropyl acetate	0.074	44.991	Methinophosphide	0.071
	44.992	Methinophosphide	0.073	42.032	Acetonitrile	0.068
	49.994	H_2_O_3_^+^	0.057	43.047	C_3_H_6_H^+^	0.056
	63.016	Dimethyl sulfide	0.057	103.068	Isopropyl acetate	0.054
	49.020	Methanethiol	0.054	73.065	Methyl Ethyl Ketone, 2-Buten-1-ol	0.053
	73.063	Methyl Ethyl Ketone, 2-Buten-1-ol	0.037	49.004	Methanethiol	0.049
	42.030	Acetonitrile	0.036	74.061	Acetamide, N-methyl-	0.041
	51.037	CH_6_O_2_H^+^	0.032	51.038	CH_6_O_2_H^+^	0.029
*B. cepacia*	43.046	C_3_H_6_H^+^	0.168	43.047	C_3_H_6_H^+^	0.201
	44.044	C_2_H_5_NH^+^	0.151	42.032	Acetonitrile	0.171
	181.007	p-Xylene	0.122	281.066	NA	0.134
	49.020	Methanethiol	0.110	47.041	(CH_5_NO)^+^	0.097
	281.053	NA	0.099	44.991	Methinophosphide	0.088
	47.039	(CH_5_NO)^+^	0.095	55.039	C_2_H_2_N_2_H^+^	0.088
	42.030	Acetonitrile	0.076	49.004	Methanethiol	0.062
	55.039	C_2_H_2_N_2_H^+^	0.072	181.014	p-Xylene	0.056
	51.037	CH_6_O_2_H^+^	0.071	44.048	C_2_H_5_NH^+^	0.052
	44.992	Methinophosphide	0.037	51.038	CH_6_O_2_H^+^	0.051
“Severe” genotype *	281.053	NA	0.545	281.066	NA	0.517
	118.066	Indole	0.097	118.070	Indole	0.122
	62.028	Carbamic acid	0.061	62.028	Carbamic acid	0.084
	57.065	1-Propene, 2-methyl-	0.059	51.038	CH_6_O_2_H^+^	0.074
	44.992	Methinophosphide	0.055	44.991	Methinophosphide	0.067
	51.037	CH_6_O_2_H^+^	0.041	43.047	C_3_H_6_H^+^	0.060
	45.994	NO_2_^+^	0.040	49.994	H_2_O_3_^+^	0.043
	49.994	H_2_O_3_^+^	0.035	47.041	(CH_5_NO)^+^	0.022
	47.039	(CH_5_NO)^+^	0.028	57.069	1-Propene, 2-methyl-	0.010
	43.046	C_3_H_6_H^+^	0.023	75.046	Methyl acetate	0.001
	75.042	Methyl acetate	0.016	45.992	NO_2_^+^	0.000
HEMT	101.050	C_5_H_8_O_2_H^+^	0.170	43.047	C_3_H_6_H^+^	0.208
	97.086	1,4-Dimethylpyrazole	0.131	103.068	Isopropyl acetate	0.152
	49.020	Methanethiol	0.119	49.004	Methanethiol	0.141
	103.072	Isopropyl acetate	0.113	58.960	NA	0.133
	69.071	1,4-Pentadiene (isoprene)	0.110	69.072	1,4-Pentadiene (isoprene)	0.108
	63.016	Dimethyl sulfide	0.097	63.017	Dimethyl sulfide	0.061
	42.030	Acetonitrile	0.085	75.046	Methyl acetate	0.057
	58.952	NA	0.070	101.043	C_5_H_8_O_2_H^+^	0.054
	43.046	C_3_H_6_H^+^	0.065	97.094	1,4-Dimethylpyrazole	0.044
	75.042	Methyl acetate	0.039	42.032	Acetonitrile	0.042

DLCO: diffusing capacity of the lungs for carbon monoxide; FEV_1_: forced expiratory volume in 1 s; FVC: forced vital capacity; FEF_75_: forced expiratory flow when 75% of FVC has been exhaled; HEMT: highly effective modulator treatment; LLN: lower limit of normal (z-score < −1.645); * Class 1, 2 or 3 of CFTR mutation [20].

## Data Availability

The data presented in this study are available on request from the corresponding author.

## Supplementary Materials

The following supporting information can be downloaded at: https://www.mdpi.com/article/10.3390/biom14091189/s1, Table S1. VOCs described in the literature as CF markers: GC-MS—gas chromatography–mass spectrometry; GC-TOF-MS—gas chromatography coupled to time-of-flight mass spectrometry; HPLC—high-performance liquid chromatography; *m*/*z*—mass-to-charge ratio; NMR metabolomics—nuclear magnetic resonance based metabolomics; PTR-MS—proton-transfer-reaction mass spectrometry; SESI-MS—secondary electrospray ionization-mass spectrometry; SIFT-MS—selected-ion flow-tube mass spectrometry; SPME-GC-MS—solid phase micro-extraction and gas chromatography with mass spectrometry; UPLC-MS—ultraperformance liquid chromatography-mass spectrometry; VOCs—volatile organic compounds. Table S2. Selected VOCs for functional and clinical endpoints in the forced expiratory maneuver and normal quiet breathing: CFTR—cystic fibrosis transmembrane conductance regulator; DLCO—diffusing capacity of the lungs for carbon monoxide; FEV_1_: forced expiratory volume in 1 s; FVC: forced vital capacity; FEF_75:_ forced expiratory flow when 75% of FVC has been exhaled; HEMT: highly effective modulator treatment; IL: IONICON library; LLN: lower limit of normal (z-score < −1.645); NA: not available; PTR-MS VMS: proton mass-spectrometry view mass calculator; * Class 1, 2 or 3 of CFTR mutation. Table S3. Demographic characteristics according to respiratory function indicators: BMI—Body mass index; CFTR: cystic fibrosis transmembrane conductance regulator; DLCO—diffusing capacity of the lungs for carbon monoxide; FEV_1_—forced expiratory volume in 1 second; FVC—forced vital capacity; FEF_75_—the forced expiratory flow when 75% of FVC has been exhaled; HEMT—highly effective modulator treatment; mMRC—Modified Medical Research Council; LLN—lower limit of normal; SD—standard deviation; * Class 1, 2 or 3 of CFTR mutation; ** Endpoints (z-score < −1.645).

## References

[1] Dickinson K.M., Collaco J.M. (2021). Cystic Fibrosis. Pediatr. Rev..

[2] McBennett K.A., Davis P.B., Konstan M.W. (2022). Increasing life expectancy in cystic fibrosis: Advances and challenges. Pediatr. Pulmonol..

[3] Block J.K., Vandemheen K.L., Tullis E., Fergusson D., Doucette S., Haase D., Berthiaume Y., Brown N., Wilcox P., Bye P. (2006). Predictors of pulmonary exacerbations in patients with cystic fibrosis infected with multi-resistant bacteria. Thorax.

[4] Filipiak W., Ager C., Troppmair J. (2017). Predicting the future from the past: Volatile markers for respiratory infections. Eur. Respir. J..

[5] Kamboures M.A., Blake D.R., Cooper D.M., Newcomb R.L., Barker M., Larson J.K., Meinardi S., Nussbaum E., Rowland F.S. (2005). Breath sulfides and pulmonary function in cystic fibrosis. Proc. Natl. Acad. Sci. USA.

[6] Barker M., Hengst M., Schmid J., Buers H.J., Mittermaier B., Klemp D., Koppmann R. (2006). Volatile organic compounds in the exhaled breath of young patients with cystic fibrosis. Eur. Respir. J..

[7] Gaisl T., Bregy L., Stebler N., Gaugg M.T., Bruderer T., García-Gómez D., Moeller A., Singer F., Schwarz E.I., Benden C.M.-L. (2018). Real-time exhaled breath analysis in patients with cystic fibrosis and controls. J. Breath Res..

[8] Robroeks C.M., van Berkel J.J., Dallinga J.W., Jöbsis Q., Zimmermann L.J., Hendriks H.J., Wouters M.F., van der Grinten C.P., van de Kant K.D., van Schooten F.J. (2010). Metabolomics of volatile organic compounds in cystic fibrosis patients and controls. Pediatr. Res..

[9] Gilchrist F.J., Razavi C., Webb A.K., Jones A.M., Spaněl P., Smith D., Lenney W. (2012). An investigation of suitable bag materials for the collection and storage of breath samples containing hydrogen cyanide. J. Breath Res..

[10] Smith D., Sovová K., Dryahina K., Doušová T., Dřevínek P., Španěl P. (2016). Breath concentration of acetic acid vapour is elevated in patients with cystic fibrosis. J. Breath Res..

[11] Španěl P., Sovová K., Dryahina K., Doušová T., Dřevínek P., Smith D. (2016). Do linear logistic model analyses of volatile biomarkers in exhaled breath of cystic fibrosis patients reliably indicate Pseudomonas aeruginosa infection?. J. Breath Res..

[12] Neerincx A.H., Geurts B.P., van Loon J., Tiemes V., Jansen J.J., Harren F.J., Kluijtmans L.A., Merkus P.J., Cristescu S.M., Buydens L.M. (2016). Detection of Staphylococcus aureus in cystic fibrosis patients using breath VOC profiles. J. Breath Res..

[13] Seidl E., Licht J.C., de Vries R., Ratjen F., Grasemann H. (2024). Exhaled Breath Analysis Detects the Clearance of Staphylococcus aureus from the Airways of Children with Cystic Fibrosis. Biomedicines.

[14] Montuschi P., Paris D., Melck D., Lucidi V., Ciabattoni G., Raia V., Calabrese C., Bush A., Barnes P.J., Motta A. (2012). NMR spectroscopy metabolomic profiling of exhaled breath condensate in patients with stable and unstable cystic fibrosis. Thorax.

[15] Zang X., Monge M.E., McCarty N.A., Stecenko A.A., Fernández F.M. (2017). Feasibility of Early Detection of Cystic Fibrosis Acute Pulmonary Exacerbations by Exhaled Breath Condensate Metabolomics: A Pilot Study. J. Proteome Res..

[16] van Horck M., Smolinska A., Wesseling G., de Winter-de Groot K., de Vreede I., Winkens B., Jöbsis Q., Dallinga J., Dompeling E., van Schooten F.J. (2021). Exhaled volatile organic compounds detect pulmonary exacerbations early in children with cystic fibrosis: Results of a one-year observational pilot study. J. Breath Res..

[17] Woollam M., Siegel A.P., Grocki P., Saunders J.L., Sanders D.B., Agarwal M., Davis M.D. (2022). Preliminary method for profiling volatile organic compounds in breath that correlate with pulmonary function and other clinical traits of subjects diagnosed with cystic fibrosis: A pilot study. J. Breath Res..

[18] Mustafina M., Silantyev A., Krasovskiy S., Chernyak A., Naumenko Z., Suvorov A., Gognieva D., Abdullaev M., Bektimirova A., Bykova A. (2024). Exhaled breath analysis in adult patients with cystic fibrosis by real-time proton mass spectrometry. Clin. Chim. Acta.

[19] Stanojevic S., Kaminsky D.A., Miller M.R., Thompson B., Aliverti A., Barjaktarevic I., Cooper B.G., Culver B., Derom E., Hall G.L. (2022). ERS/ATS technical standard on interpretive strategies for routine lung function tests. Eur. Respir. J..

[20] Veit G., Avramescu R.G., Chiang A.N., Houck S.A., Cai Z., Peters K.W., Hong J.S., Pollard H.B., Guggino W.B., Balch W.E. (2016). From CFTR biology toward combinatorial pharmacotherapy: Expanded classification of cystic fibrosis mutations. Mol. Biol. Cell.

[21] Montuschi P., Paris D., Montella S., Melck D., Mirra V., Santini G., Mores N., Montemitro E., Majo F., Lucidi V. (2014). Nuclear magnetic resonance-based metabolomics discriminates primary ciliary dyskinesia from cystic fibrosis. Am. J. Respir. Crit. Care Med..

[22] Filipiak W., Sponring A., Filipiak A., Ager C., Schubert J., Miekisch W., Amann A., Troppmair J. (2010). TD-GC-MS analysis of volatile metabolites of human lung cancer and normal cells in vitro. Cancer Epidemiol. Biomark. Prev..

[23] van Mastrigt E., Reyes-Reyes A., Brand K., Bhattacharya N., Urbach H.P., Stubbs A.P., de Jongste J.C., Pijnenburg M.W. (2016). Exhaled breath profiling using broadband quantum cascade laser-based spectroscopy in healthy children and children with asthma and cystic fibrosis. J. Breath Res..

[24] Martinez-Medina M., Denizot J., Dreux N., Robin F., Billard E., Bonnet R., Darfeuille-Michaud A., Barnich N. (2014). Western diet induces dysbiosis with increased E coli in CEABAC10 mice, alters host barrier function favouring AIEC colonisation. Gut.

[25] Antus B., Barta I., Csiszer E., Kelemen K. (2012). Exhaled breath condensate pH in patients with cystic fibrosis. Inflamm. Res..

[26] Phillips M., Cataneo R.N., Greenberg J., Grodman R., Gunawardena R., Naidu A. (2003). Effect of oxygen on breath markers of oxidative stress. Eur. Respir. J..

[27] Papaefstathiou E., Bezantakos S., Stylianou M., Biskos G., Agapiou A. (2020). Comparison of particle size distributions and volatile organic compounds exhaled by e-cigarette and cigarette users. J. Aerosol. Sci..

[28] Enderby B., Smith D., Carroll W., Lenney W. (2009). Hydrogen cyanide as a biomarker for Pseudomonas aeruginosa in the breath of children with cystic fibrosis. Pediatr. Pulmonol..

[29] Pabary R., Huang J., Kumar S., Alton E.W., Bush A., Hanna G.B., Davies J.C. (2016). Does mass spectrometric breath analysis detect Pseudomonas aeruginosa in cystic fibrosis?. Eur. Respir. J..

[30] Scott J., Sueiro-Olivares M., Ahmed W., Heddergott C., Zhao C., Thomas R., Bromley M., Latgé J.P., Krappmannm S., Fowler S. (2019). Pseudomonas Aeruginosa-Derived Volatile Sulfur Compounds Promote Distal Aspergillus Fumigatus Growth and a Synergistic Pathogen-Pathogen Interaction That Increases Pathogenicity in Co-infection. Front Microbiol..

[31] Natal Jorge R.M., Tavares J.M.R., Pinotti Barbosa M., Slade A.P. (2011). Technology and Medical Sciences.

[32] Eberl L., Tümmler B. (2004). Pseudomonas aeruginosa and Burkholderia cepacia in cystic fibrosis: Genome evolution, interactions and adaptation. Int. J. Med. Microbiol..

[33] Buszewski B., Ulanowska A., Ligor T., Denderz N., Amann A. (2009). Analysis of exhaled breath from smokers, passive smokers and non-smokers by solid-phase microextraction gas chromatography/mass spectrometry. Biomed. Chromatogr..

[34] Montuschi P., Lucidi V., Paris D., Montemitro E., Shohreh R., Mores N., Melck D., Santini G., Majo F., Motta A. (2018). Metabolomic Analysis by Nuclear Magnetic Resonance Spectroscopy as a New Approach to Understanding Inflammation and Monitoring of Pharmacological Therapy in Children and Young Adults with Cystic Fibrosis. Front. Pharmacol..

