# Supplementary material for: Identification of Exhaled Metabolites Correlated with Respiratory Function and Clinical Features in Adult Patients with Cystic Fibrosis by Real-Time Proton Mass Spectrometry
Source: Biomolecules. 2024 Sep 21;14(9):1189. doi: 10.3390/biom14091189 (PMC11430581; doi:10.3390/biom14091189)
Supplement: Supplementary file 1 [file biomolecules-14-01189-s001.zip › biomolecules-3205092-supplementary.pdf]

Supplementary materials

Table S1. VOCs described in the literature as cystic fibrosis markers

Study	Analytical method	VOCs	Formula	SMILES	Adduct [M+H] +, Da	Class
McGrath et al. (2000) [1]	GC-MS	Isoprene	C5H8	CC(=C)C=C	71.070	Cyclic hydrocarbons. Terpenes
Paredi et al. (2000) [2]	GC-MS	Ethane	C2H6	CC	31.054	Hydrocarbons. Alkanes
Kamboures et al. (2005) [3]	gas analytical system	Carbon disulfide	CS2	C(=S) =S	76.951	Sulfur compound
		Carbonyl sulfide	COS	C(=O)=S	60.9743	Sulfur compound
		Dimethyl sulfide	C2H6S	CSC	63.0263	Sulfur compound
Barker et al. (2006) [4]	GC-MS	Dimethyl sulfide	C2H6S	CSC	64.0263	Sulfur compound
		Pentane	C5H12	CCCCC	73.101	Hydrocarbons. Alkanes
Enderby et al. (2009) [5]	SIFT-MS	Hydrogen cyanide	CHN	C#N	29.018	Organonitrogen compounds
Robroeks et al. (2010) [6]	GC-MS	2-Buten-1-ol	C4H8O	CC=CCO	73.065	Alcohols
		2-Octene	C8H16	CCCCCC=CC	113.133	Hydrocarbons. Alkenes
		3,3-Dimethylhexane	C8H18	CCCC(C)(C)CC	115.148	Hydrocarbons. Alkanes
		cis-3-Octene	C8H16	CCCCC=CCC	113.133	Hydrocarbons. Alkenes
		Benzothiazole	C7H5NS	C1=CC=C2C(=C1)N=CS2	136.022	Sulfur compound
		N-Methylisobutylamine	C5H13N	CC(C)CNC	88.112	Organonitrogen compound.
		4-Methylbenzaldehyde	C8H8O	CC1=CC=C(C=C1)C=O	121.065	Cyclic hydrocarbons. Benzene derivatives

van Berkel et al. (2010) [7]	GC-TOF-MS	Pentane	C5H13	CCCCC	74.101	Hydrocarbons. Alkanes
		trans-2-Nonenal	C9H16O	CCCCCCC=CC=O	141.127	Aldehydes
		Azulene	C10H8	C1=CC=C2C=CC=C2C=C1	129.070	Cyclic hydrocarbons
		Eicosane	C20H42	CCCCCCCCCCCCCCCCCCCC	283.336	Hydrocarbons. Alkanes
		1-Aminobutan-2-ol	C4H11NO	CCC(CN)O	90.091	Amino alcohols. Organonitrogen compound
Scott-Thomas et al. (2010) [8]	GS-MS	2'-Aminoacetophenone	C8H9NO	CC(=O)C1=CC=CC=C1N	136.076	Ketones
Shestivska et al. (2011) [9]	SIFT-MS	Methyl thiocyanate	C2H3NS	CSC#N	74.006	Organonitrogen compound.
Gramacho et al. (2011) [10]	SPME-GC-MS	Dichloromethane	CH2Cl2	C(Cl)Cl	84.961	Hydrocarbons
		Chloroform	CHCl3	C(Cl)(Cl)Cl	118.922	Hydrocarbons
		Bromochloromethane	CH2BrCl	C(Cl)Br	128.910	Hydrocarbons
		Methylene Chloride	CH2Cl2	C(Cl)Cl	84.961	
		4-Methyl-2-pentanone	C6H12O	CC(C)CC(=O)C	101.10	Ketones
		Acetonitrile	CH3CN	CC#N	42.034	Organonitrogen compounds
		Isopropyl Alcohol	C3H8O	CC(C)O	61.065	Alcohols
		Pentane	C5H14	CCCCC	75.101	Hydrocarbons. Alkanes
		Toluene	C7H8	CC1=CC=CC=C1	93.070	Cyclic hydrocarbons. Benzene Derivatives
		Ethanol	C2H6O	CCO	47.049	Alcohols
		Acetone	C3H6O	CC(=O)C	59.049	Ketones
Gilchrist et al.	SIFT-MS	Hydrogen cyanide	CHN	C#N	30.018	Organonitrogen compounds

(2012) [11]						
Montuschi et al. (2012) [12]	NMR metabolomics	Ethanol	C2H6O	CCO	51.049	Alcohols
		Isopropyl alcohol	C3H8O	CC(C)O	61.065	Alcohols
		Acetone	C3H6O	CC(=O)C	61.049	Ketones
		Methanol	CH4O	CO	36.034	Alcohols
White et al. (2013) [13]	PTR-TOF-MS	Isoprene	C5H8	CC(=C)C=C	69.070	Cyclic hydrocarbons. Terpenes
		Methanol	CH4O	CO	35.034	Alcohols
Montuschi et al. (2014) [14]	NMR metabolomics	Methanol	CH4O	CO	37.034	Alcohols
		Acetoin	C4H8O3	CC(C(=O)C)O	105.055	Ketones
Gilchrist et al. (2015) [15]	SIFT-MS	Hydrogen cyanide	CHN	C#N	31.018	Organonitrogen compounds
Antus et al. (2015) [16]	HPLC	Malonaldehyde	C3H4O3	C(C=O) C=O	74.028	Aldehydes
Smith et al. (2016) [17]	SIFT-MS	Acetic acid	C2H4O2	CC(=O)O	61.028	Organic acid
		Isoprene	C5H8	CC(=C)C=C	70.070	Cyclic hydrocarbons. Terpenes
Bos et al. (2016) [18]	GS-MS	2,3-Butanedione	C4H6O2	CC(=O) C(=O) C	87.044	Ketones
		Acetaldehyde	C2H4O	CC=O	45.034	Aldehydes
		Ethanol	C2H6O	CCO	48.049	Alcohols
		Hydrogen Cyanide	CHN	C#N	28.018	Organonitrogen compounds
		Methanol	CH4O	CO	33.034	Alcohols
Neerincx et al. (2016) [19]	GS-MS	1,4-Pentadiene	C5H8	C=CCC=C	69.070	Hydrocarbons
		Acetoin	C4H8O2	CC(C(=O)C)O	89.060	Ketones
		2-Methylnaphthalene	C11H10	CC1=CC2=CC=CC=C2C=C1	143.086	Cyclic hydrocarbons

		Acetone	C3H6O	CC(=O)C	60.049	Ketones
		Ethanol	C2H6O	CCO	49.049	Alcohols
		Hexanal	C6H12O	CCCCCC=O	101.096	Aldehydes
		Isopropyl myristate	C17H34O2	CCCCCCCCCCCCCC(=O)OC(C)C	271.263	Esters
		Undecane	C11H24	CCCCCCCCCCC	157.195	Hydrocarbons. Alkanes
Španěl et al. (2016) [20]	SIFT-MS	Malonaldehyde	C3H4O2	C(C=O)C=O	73.028	Aldehydes
		Methanol	CH4O	CO	34.034	Alcohols
		Phenol	C6H6O	C1=CC=C(C=C1)O	95.049	Cyclic hydrocarbons. Benzene derivatives
		acetoin	C4H8O2	CC(C(=O)C)O	89.060	Ketones
van Mastrigt et al. (2016) [21]	laser-based spectroscopy	1-(3-Methoxypropoxy) propan-1-ol	C7H16O3	CCC(O)OCCCOC	149.117	Hydrocarbons
		Dioxane	C4H8O2	C1COCCO1	89.060	Cyclic hydrocarbons
		2-Ethoxyethyl acetate	C6H12O3	CCOCCOC(=O)C	133.086	Alcohols
		Butyl acetate	C6H12O2	CCCCOC(=O)C	117.091	Esters
		Dichloronitromethane	CHCl2NO2	C([N+](=O)[O-])(Cl)Cl	129.946	Nitro compounds
		Dimethyl carbonate	C3H6O3	COC(=O)OC	91.039	Esters
		Ethyl acetate	C4H8O2	CCOC(=O)C	89.060	Esters
		Ethyl acrylate	C5H8O2	CCOC(=O)C=C	101.060	Esters
		Ethyl butyrate	C6H12O2	CCCC(=O)OCC	117.091	Esters
		Isobutyl acetate	C6H12O2	CC(C)COC(=O)C	117.091	Esters
		Isopropyl acetate	C5H10O2	CC(C)OC(=O)C	103.075	Organic acid

		Methyl acetate	C3H6O2	CC(=O)OC	75.044	Esters
Pabary et al. (2016) [22]	SIFT-MS	2'-Aminoacetophenone	C8H9NO	CC(=O)C1=CC=CC=C1N	136.076	Ketones
		1-Butanol	C4H10O	CCCCO	75.080	Alcohols
		Dimethyl disulfide	C2H6S2	CSSC	94.998	Sulfur compound
Zang et al. (2017) [23]	UPLC-MS	trans-4-hydroxycyclohexylcarboxylic acid	C7H12O3	C1CC(CCC1C(=O)O)O	145.086	Organic acid
		Lactic acid	C3H6O3	CC(C(=O)O)O	91.039	Organic acid
Gaisl et al. (2018) [24]	SESI-MS, liquid chromatography	Benzothiazole	C7H5NS	C1=CC=C2C(=C1) N=CS3	137.022	Sulfur compound
		Docosahexaenoic acid (DHA)	C22H32O2	CCC=CCC=CCC=CCC=CCC=CCC=CCCC (= O) O	329.248	Organic acid
		2-Hydroxyoctanoic acid	C8H16O3	CCCCCCC(C(=O) O) O	161.117	Organic acid
		2-Oxohexanoic acid	C6H10O3	CCCCC(=O) C(=O) O	131.070	Organic acid
Zang et al. (2020) [25]	UPLC-MS	Acetic acid	C2H4O3	CC(=O)O	62.0285	Organic acid
		Carnitine	C7H15NO4	C[N+](C)(C)CC(CC(=O)[O-])O	163.113	Esters
		trans-4-hydroxycyclohexylcarboxylic acid	C7H12O4	C1CC(CCC1C(=O)O)O	161.081	Organic acid
		Lactic acid	C3H6O4	CC(C(=O)O)O	107.034	Organic acid
		Malic acid	C4H6O5	C(C(C(=O)O)O)C(=O)O	135.029	Organic acid
		L-Pyroglutamic acid	C5H7NO3	C1CC(=O)NC1C(=O)O	130.050	Organic acid
van Horck et al.	GC-TOF-MS	m-Xylene	C8H10	CC1=CC(=CC=C1)C	107.086	Cyclic hydrocarbons. Benzene

(2021) [26]						derivatives
		2,4-dimethyl-1-heptene	C_9H_{18}	CCCC(C)CC(=C)C	127.148	Hydrocarbons. Alkenes
		3-methyl-2-butanone	$C_5H_{10}O$	CC(C)C(=O)C	87.080	Ketones
		Camphene	$C_{10}H_{16}$	CC1(C2CCC(C2)C1=C)C	137.132	Cyclic hydrocarbons. Terpenes
		p-benzoquinone	$C_6H_4O_2$	C1=CC(=O)C=CC1=O	109.028	Ketones
		Pentadecane	$C_{15}H_{32}$	CCCCCCCCCCCCCCC	213.258	Hydrocarbons. Alkanes
		Tetradecanal	$C_{14}H_{28}O$	CCCCCCCCCCCCCC=O	213.221	Aldehydes
Kos et al. (2021) [27]	GC-MS	Methyl ethyl ketone	C_4H_8O	CCC(=O)C	73.065	Ketones
		2-Pentanone	$C_5H_{10}O$	CCCC(=O)C	87.080	Ketones
		Ethyl acetate	$C_4H_8O_3$	CCOC(=O)C	90.060	Esters
		2,4-Dimethylheptane	C_9H_{20}	CCCC(C)CC(C)C	129.1638257	Hydrocarbons. Alkenes
		Limonene	$C_{10}H_{16}$	CC1=CCC(CC1)C(=C)C	137.1325255	Cyclic hydrocarbons. Terpenes
		Toluene	C_7H_9	CC1=CC=CC=C2	94.06992529	Cyclic hydrocarbons. Benzene derivatives
Woollam et al. (2022) [28]	GC-MS	2,2,4-Trimethyl-1,3-pentanediol	$C_8H_{18}O_2$	CC(C)C(C(C)(C)CO)O	147.1380049	Organic acid
		3,7-Dimethyldecane	$C_{12}H_{26}$	CCCC(C)CCCC(C)CC	171.2107759	Hydrocarbons. Alkanes
		5-Methyltridecane	$C_{14}H_{30}$	CCCCCCCCC(C)CCCC	199.242076	Hydrocarbons
		1,2,4,5-Tetramethylbenzene	$C_{10}H_{14}$	CC1=CC(=C(C=C1C)C)C	135.1168755	Cyclic hydrocarbons. Benzene derivatives
Mani-Varnosfaderani et al. (2022) [29]	GC-TOF-MS	Cyclooctane, 1,4-dimethyl-, cis-	$C_{10}H_{20}$	CC1CCCCC(CC1)C	141.1638257	Cyclic hydrocarbons
		5-Methylheptylbenzene	$C_{14}H_{22}$	CCC(C)CCCCC1=CC=CC=C1	191.1794757	Cyclic hydrocarbons. Benzene derivatives

		2,2,4,6,6-Pentamethylheptane	C12H26	CC(CC(C)(C)C)CC(C)(C)C	171.2107759	Hydrocarbons. Alkenes
		2,3,5,8-Tetramethyldecane	C14H30	CCC(C)CCC(C)CC(C)C(C)C	199.242076	Hydrocarbons. Alkanes
		2,3-Dimethyl-3-heptene	C9H18	CCCC=C(C)C(C)C	127.1481756	Hydrocarbons. Alkenes
		2,6,10,14-Tetramethylheptadecane	C21H44	CCCC(C)CCCC(C)CCCC(C)CCCC(C)C	297.3516265	Hydrocarbons. Alkanes
		2,6-Bis(1,1-dimethylethyl)-4-(1-oxopropyl) phenol	C17H26O2	CCC(=O)C1=CC(=C(C(=C1)C(C)(C)C)O)C(C)(C)C	263.2006051	Protease-activated receptor
		2-Bromododecane	C12H25Br	CCCCCCCCCCC(C)Br	249.1212884	Hydrocarbons
		2-Methylundecane-2-thiol	C12H26S	CCCCCCCCCC(C)(C)S	203.182847	Sulfur compound
		(Aminomethyl)cyclopropane	C4H9N	C1CC1CN	72.08082433	Organonitrogen compound.
		Ethanol	C2H6O	CCO	50.04918985	Alcohols
		1-Tridecanol	C13H28O	CCCCCCCCCCCCCO	201.2213406	Alcohols
		Tridecane	C13H28	CCCCCCCCCCCCC	185.2264259	Hydrocarbons. Alkanes
Weber et al. (2022) [30]	SESI-MS	4-Acetamidobutyric acid	C6H11NO3	CC(=O)NCCCC(=O)O	146.0812182	Organic acid
		5-Diazouracil	C4H2N4O2	C1=C(C(=O)NC(=N1)[O-])[N+]#N	139.0251004	Organonitrogen compounds
		11-Oxoundecanoic acid	C11H20O3	C(CCCCC=O)CCCCC(=O)O	201.1485695	Organic acid
		L-2-Aminoadipic acid	C6H11NO4	C(CC(C(=O)O)N)CC(=O)O	162.0761329	Organic acid
		2-Decenal	C10H18O	CCCCCCCC=CC=O	155.1430902	Aldehydes
		2-Methylbutyrylcarnitine	C12H23NO4	CCC(C)C(=O)OC(CC(=O)[	246.1700333	Esters

				O-])C[N+](C)(C)C		
		3-Hydroxyoctanoylcarnitine	C15H29NO5	CCCCCC(CC(=O)OC(CC(=O)[O-])C[N+](C)(C)C)O	304.2118981	Esters
		7-Oxoheptanoic acid	C7H12O3	C(CCC=O)CCC(=O)O	145.0859693	Organic acid
		Butenylcarnitine	C11H19NO4	CC=CC(=O)OC(CC(=O)[O-])C[N+](C)(C)C	230.1387331	Esters
		Butyrylcarnitine	C11H21NO4	CCCC(=O)OC(CC(=O)[O-])C[N+](C)(C)C	232.1543832	Esters
		Carnitine	C7H15NO3	C[N+](C)(C)CC(CC(=O)[O-])O	162.1125184	Esters
		3-Dehydrocarnitine	C7H13NO3	C[N+](C)(C)CC(=O)CC(=O)[O-]	160.0968683	Esters
		Diethanolamine	C4H11NO2	C(CO)NCCO	106.0863036	Amino alcohols
		Dodecamethylcyclohexasiloxane	C12H36O6Si6	C[Si]1(O[Si](O[Si](O[Si](O[Si](O[Si](O1)(C)C)(C)C)(C)C)(C)C)(C)C)C	445.1200731	
		Evocarpine	C23H33NO	CCCCC=CCCCCCCCC1=CC(=O)C2=CC=CC=C2N1C	340.2635397	Organonitrogen compound
		Caproic Acid	C6H12O2	CCCCCC(=O)O	117.0910547	Organic acid
		Hydroxyisovaleroyl carnitine	C12H23NO5	CC(C)CC(=O)OC(CC(=O)[O-])(C[N+](C)(C)C)O	262.1649479	Esters
		Hydroxypropionylcarnitine	C10H19NO5	C[N+](C)(C)CC(CC(=O)[O-])OC(=O)CCO	234.1336477	Esters
		Octanoylcarnitine	C15H29NO4	CCCCCCCC(=O)OC(CC(=O)[O-])C[N+](C)(C)C	288.2169834	Esters

		Trimethylamine	C3H9N	CN(C)C	60.08082433	Organonitrogen compound
		Xanthine	C5H4N4O2	C1=NC2=C(N1)C(=O)NC(=O)N2	153.0407504	Alkaloids
		8-Amino-7-oxononanoic acid	C9H17NO3	CC(C(=O)CCCCCC(=O)O)N	188.1281684	Organic acid
		O-acetylcarnitine	C9H18NO4+	CC(=O)OC(CC(=O)O)C[N+](C)(C)C	204.1230345	Esters
		Glyceric acid	C3H6O4	C(C(C(=O)O)O)O	107.0339337	Organic acid
		2-Octenal	C8H14O	CCCCCC=CC=O	127.1117901	Aldehydes
		Propenoylcarnitine	C10H17NO4	C[N+](C)(C)CC(CC(=O)[O-])OC(=O)C=C	216.1230831	Esters
		4-Hydroxydecenal	C10H18O2	CCCCCCC(C=CC=O)O	171.1380049	Aldehydes
		9-Oxononanoic acid	C9H16O3	C(CCCC=O)CCCC(=O)O	173.1172694	Organic acid
		O-acryloyl-L-carnitine	C10H17NO4	C[N+](C)(C)CC(CC(=O)[O-])OC(=O)C=C	216.1230831	Esters
		Glycolic acid	C2H4O3	C(C(=O)O)O	77.02336902	Organic acid
		Octenoylcarnitine	C15H27NO4	CCCCCC=CC(=O)C(CC(=O)[O-])(C[N+](C)(C)C)O	286.2013334	Esters
		Propionylcarnitine	C10H19NO4	CCC(=O)OC(CC(=O)[O-])C[N+](C)(C)C	218.1387331	Esters
		4-Hydroxyheptenal	C7H12O2	CCCC(C=CC=O)O	129.0910547	Aldehydes
		Sideretin	C10H8O6	COC1=C(C(=C2C(=C1O)C=CC(=O)O2)O)O	225.039413	Organic heteropolycyclic compound
		4-Hydroxyhexenal	C6H10O2	CCC(C=CC=O)O	115.0754046	Aldehydes

		Tiglylcarnitine	C12H21NO4	CC=C(C)C(=O)OC(CC(=O)[O-])C[N+](C)(C)C	244.1543832	Esters
		4-Hydroxy-2-octenal	C8H14O2	CCCCC(C=CC=O)O	143.1067047	Aldehydes
		4-Hydroxy-2-tetradecenal	C14H26O2	CCCCCCCCCCC(C=CC=O)O	227.2006051	Aldehydes
Mustafina et al. (2024) [31]	PTR-TOF-MS	Indole	C8H7N	C1=CC=C2C(=C1)C=CN2	118.06513	Heterocyclic compounds
		Phenol	C6H6O	C1=CC=C(C=C1)O	96.04918985	Cyclic hydrocarbons. Benzene derivatives
		Dimethyl sulfide	C2H6S	CSC	65.0263464	Sulfur compound

GC-MS – Gas chromatography–mass spectrometry; GC-TOF-MS – Gas chromatography coupled to time-of-flight mass spectrometry; HPLC – High-performance liquid chromatography; m/z – Mass-to-charge ratio; NMR metabolomics – Nuclear magnetic resonance based metabolomics; PTR-MS – Proton-transfer-reaction mass spectrometry; SESI-MS – Secondary electrospray ionization-mass spectrometry; SIFT-MS – Selected-ion flow-tube mass spectrometry; SPME-GC-MS – Solid phase micro-extraction and gas chromatography with mass spectrometry; UPLC-MS – Ultraperformance liquid chromatography-mass spectrometry; VOCs – Volatile organic compounds.

Table S2. Selected VOCs for functional and clinical endpoints in the forced expiratory maneuver and the normal quiet breathing

Endpoint	VOC name	m/z	Error, ppm	Annotation method	Endpoint	VOC name	m/z	Error, ppm	Annotation method
FEV_1/FVC < LLN	C2H5NH+	44.044	-1.590	IL	*S. aureus*	C3H6H+	43.046	-190.584	IL
	Methinophosphide	44.992	-111.476	PTR-MS VMS		C2H5NH+	44.044	-11.590	IL
	CH6O2H+	51.037	-135.287	IL		Methinophosphide	44.992	-111.476	PTR-MS VMS
	Acetic acid	61.033	81.816	[17,25]		(CH5NO)+	47.041	51.333	IL
	Carbamic acid	62.028	75.609	PTR-MS VMS		Methanethiol	49.004	-129.518	PTR-MS VMS
	Methyl acetate	75.042	-2.559	[21]		CH6O2H+	51.037	-135.287	IL

	Indole	118.066	7.503	[31]		C2H2N2H+	55.039	174.810	IL
	p-Xylene	181.007	22.477	[26]		NA	58.960	NA	NA
						1,3-Cyclopentadiene	67.056	9.403	PTR-MS VMS
						Cyclopropane, (1-methylethenyl)-	83.086	-12.707	PTR-MS VMS
						Lactic acid	91.055	157.126	[23,25]
FEF_{75} < LLN	C3H6H+	43.046	-190.584	IL		Phenol	95.054	114.526	[20,31]
	Methinophosphide	44.992	-111.476	PTR-MS VMS		4-Methylbenzaldehyde	121.094	184.865	[6]
	NO2+	45.994	37.584	IL	*P. aeruginosa*	Acetonitrile	42.032	-101.284	[10]
	(CH5NO)+	47.039	51.333	IL		C3H6H+	43.046	-190.584	IL
	Methoxyamine	48.044	-18.177	PTR-MS VMS		C2H5NH+	44.044	-11.590	IL
	Methanethiol	49.004	-129.518	PTR-MS VMS		Methinophosphide	44.992	-111.476	PTR-MS VMS
	H2O3+	49.994	-127.757	IL		(CH5NO)+	47.039	51.333	IL
	CH6O2H+	51.037	-135.287	IL		Methanethiol	49.004	-129.518	PTR-MS VMS
	Carbamic acid	62.028	75.609	PTR-MS VMS		H2O3+	49.994	-127.757	IL
	1,3-Pentadiene	65.046	94.306	PTR-MS VMS		CH6O2H+	51.037	-135.287	IL
	Methyl acetate	75.042	-2.559	[21]		NA	58.960	NA	NA
	Indole	118.066	7.503	[31]		Dimethyl sulfide	63.017	-156.752	[3,4,31]
						Methyl ethyl ketone, 2-buten-1-ol	73.065	-23.355	[27]

						Acetamide, N-methyl-	74.061	-30.340	PTR-MS VMS
						Isopropyl acetate	103.068	-33.174	[21]
DLCO < LLN	Acetonitrile	42.032	-36.344	[10]		Indole	118.066	7.503	[31]
	Methanethiol	49.004	-129.518	PTR-MS VMS	*B. cepacia*	Acetonitrile	42.032	-101.284	[10]
	H2O3+	49.994	-127.757	IL		C3H6H+	43.046	-190.584	IL
	1,3-Pentadiene	65.046	94.306	PTR-MS VMS		C2H5NH+	44.044	-11.590	IL
	2,3-Dimethyl-2-butene	85.095	-68.796	PTR-MS VMS		Methinophosphide	44.992	-111.476	PTR-MS VMS
	2-Pentanone (3-methyl-2-butanone)	87.078	-34.042	[27]		(CH5NO)+	47.039	51.333	IL
	C5H8O2H+	101.043	-164.825	IL		Methanethiol	49.004	-129.518	PTR-MS VMS
	3-Nitrobenzonitrile	149.036	6.111	PTR-MS VMS		CH6O2H+	51.037	-135.287	IL
						C2H2N2H+	55.039	174.810	IL
						p-Xylene	181.007	22.477	PTR-MS VMS
						NA	281.066	NA	NA
Severe genotype*	C3H6H+	43.046	-190.584	IL	HEMT	Acetonitrile	42.032	-36.344	[10]
	Methinophosphide	44.992	-111.476	PTR-MS VMS		C3H6H+	43.046	-190.584	IL
	NO2+	45.994	37.584	IL		Methanethiol	49.004	-129.518	PTR-MS VMS
	(CH5NO)+	47.039	51.333	IL		NA	58.960	NA	NA
	H2O3+	49.994	-127.757	IL		Dimethyl sulfide	63.017	-156.752	[3,4,31]
	CH6O2H+	51.037	-135.287	IL		1,4-Pentadiene (isoprene)	69.072	12.389	[1,13,20]
	1-Propene, 2-	57.070	-8.044	PTR-MS		Methyl acetate	75.042	-26.559	[21]

	methyl-			VMS					
	Carbamic acid	62.028	75.609	PTR-MS VMS		1,4-Dimethylpyrazole	97.094	104.472	PTR-MS VMS
	Methyl acetate	75.042	-26, .559	[21]		C5H8O2H+	101.043	-164.825	IL
	Indole	118.066	7.503	[31]		Isopropyl acetate	103.068	-33.174	[21]
	NA	281.066	NA	NA					

CFTR — cystic fibrosis transmembrane conductance regulator; DLCO — diffusing capacity of the lungs for carbon monoxide; FEV_1 — forced expiratory volume in 1 second; FVC — forced vital capacity; FEF_{75} — the forced expiratory flow when 75% of FVC has been exhaled; HEMT — highly effective modulator treatment; IL — IONICON library; LLN — lower limit of normal (z-score < -1.645); NA — not available; PTR-MS VMS — proton mass-spectrometry view mass calculator; *Class 1, 2 or 3 of CFTR mutation [32]

Table S3. Demographic characteristics according to respiratory function indicators

	FVC < LLN**	FEV_1/FVC < LLN**	FEF_{75} < LLN**	DLCO < LLN**
[N (%)]	58 (56.9%)	81 (79.4%)	84 (82.4%)	39 (38.2%)
Age, years [Mean (SD)]	27.1 ± 6.8	24.1±4.2	26.0±6.2	28.3±6.6
Gender – Males [N (%)]	24 (41.4%)	16 (19.8%)	38 (45.2%)	17 (43.6%)
BMI kg·m2 [Mean (SD)]	19.7±2.9	19.7±2.6	19.4±2.6	19.2±2.4
mMRC, score	1.0±0.6	1.2±0.6	1.1±0.6	1.5±0.7
Age of diagnosis, years [Mean (SD)]	7.7±7.5	4.7±4.2	7.0±7.0	9.1±8.4
"Severe" genotype*	11 (81.0%)	73 (90.1%)	58 (69.0%)	28 (71.8%)
Culture positive [N (%)]				
Staphylococcus aureus	27 (46.6%)	12 (14.8%)	31 (36.9%)	14 (35.9%)
Pseudomonas aeruginosa	35 (60.3%)	23 (28.4%)	50 (59.5%)	20 (51.9%)
Burkholderia cepacia	4 (6.9%)	5 (6.2%)	7 (8.3%)	3 (7.7%)
HEMT [N (%)]	12 (20.7%)	21 (25.9%)	22 (26.2%)	14 (35.9%)

BMI — Body mass index, CFTR — cystic fibrosis transmembrane conductance regulator, DLCO — diffusing capacity of the lungs for carbon

monoxide, FEV_1 — forced expiratory volume in 1 second, FVC — forced vital capacity, FEF_{75} — the forced expiratory flow when 75% of FVC has been exhaled, HEMT — highly effective modulator treatment; mMRC — Modified Medical Research Council, LLN — lower limit of normal, SD — standard deviation
*Class 1, 2 or 3 of CFTR mutation [32]; **Endpoints (z-score < -1.645).

References

1. McGrath, L.T.; Patrick, R.; Mallon, P.; Dowey, L.; Silke, B.; Norwood, W.; Elborn, S. Breath isoprene during acute respiratory exacerbation in cystic fibrosis. *Eur. Respir. J.* **2000** *16(6)*, 1065–1069. doi: 10.1034/j.1399-3003.2000.16f08.x.
2. Paredi, P.; Kharitonov, S.A.; Leak, D.; Shah, P.L.; Cramer, D.; Hodson, M.E.; Barnes, P.J. Exhaled ethane is elevated in cystic fibrosis and correlates with carbon monoxide levels and airway obstruction. *Am. J. Respir. Crit. Care Med.* **2000** *161(4 Pt 1)*, 1247–1251. doi: 10.1164/ajrccm.161.4.9906122.
3. Kamboures, M.A.; Blake, D.R.; Cooper, D.M.; Newcomb, R.L.; Barker, M.; Larson, J.K.; Meinardi, S.; Nussbaum, E.; Rowland, F.S. Breath sulfides and pulmonary function in cystic fibrosis. *Proc. Natl. Acad. Sci. USA* **2005**, *102(44)*, 15762–15767. doi: 10.1073/pnas.0507263102.
4. Barker, M.; Hengst, M.; Schmid, J.; Buers, H.J.; Mittermaier, B.; Klemp, D.; Koppmann, R. Volatile organic compounds in the exhaled breath of young patients with cystic fibrosis. *Eur. Respir. J.* **2006** *27(5)*, 929–936. doi: 10.1183/09031936.06.00085105.
5. Enderby, B.; Smith, D.; Carroll, W.; Lenney, W. Hydrogen cyanide as a biomarker for Pseudomonas aeruginosa in the breath of children with cystic fibrosis. *Pediatr. Pulmonol.* **2009** *44(2)*, 142–147. doi: 10.1002/ppul.20963.
6. Robroeks, C.M.; van Berkel, J.J.; Dallinga, J.W.; Jöbsis, Q.; Zimmermann, L.J.; Hendriks, H.J.; Wouters, M.F.; van der Grinten, C.P.; van de Kant, K.D.; van Schooten, F.J.; Dompeling, E. Metabolomics of volatile organic compounds in cystic fibrosis patients and controls. *Pediatr. Res.* **2010** *68(1)*, 75–80. doi: https://doi.org/10.26481/dis.20101103jb10.1002/rcm.5146.
7. van Berkel, J.J.B.N. There's something in the air: Volatile organic compounds in exhaled breath in pulmonary diseases, Doctoral Thesis, Doctor of Philosophy, Maastricht University, Maastricht. 2010. https://doi.org/10.26481/dis.20101103jb.
8. Scott-Thomas, A.J.; Syhre, M.; Pattemore, P.K.; Epton, M.; Laing, R.; Pearson, J.; Chambers, S.T. 2-Aminoacetophenone as a potential breath biomarker for Pseudomonas aeruginosa in the cystic fibrosis lung. *BMC Pulm. Med.* **2010** *10*, 56. doi: 10.1186/1471-2466-10-56.
9. Shestivska, V.; Nemec, A.; Dřevínek, P.; Sovová, K.; Dryahina, K.; Spaněl, P. Quantification of methyl thiocyanate in the headspace of Pseudomonas aeruginosa cultures and in the breath of cystic fibrosis patients by selected ion flow tube mass spectrometry. *Rapid Commun. Mass Spectrom*. **2011** *25(17)*, 2459–2467. doi: 10.1002/rcm.5146.
10. Natal Jorge, R.M.; Tavares, J.M.R.; Pinotti Barbosa, M.; Slade, A.P., Eds.; *Technology and Medical Sciences*, 1st ed.; CRC Press, 2011. https://doi.org/10.1201/b11330.
11. Gilchrist, F.J.; Razavi, C.; Webb, A.K.; Jones, A.M.; Spaněl, P.; Smith, D.; Lenney, W. An investigation of suitable bag materials for the collection and storage of breath samples containing hydrogen cyanide. *J. Breath Res.* **2012** *6(3)*, 036004. doi: 10.1088/1752-7155/6/3/036004.
12. Montuschi, P.; Paris, D.; Melck, D.; Lucidi, V.; Ciabattoni, G.; Raia, V. Calabrese C, Bush A, Barnes PJ, Motta A. NMR spectroscopy metabolomic profiling of exhaled breath condensate in patients with stable and unstable cystic fibrosis. *Thorax* **2012** *67(3)*, 222–228. doi: 10.1136/thoraxjnl-2011-200072.
13. White, I.R.; Willis, K.A.; Whyte, C.; Cordell, R.; Blake, R.S.; Wardlaw, A.J.; Rao, S.; Grigg, J.; Ellis, A.M.; Monks, P.S. Real-time multi-marker measurement of organic compounds in human breath: towards fingerprinting breath. *J. Breath Res.* **2013** *7(1)*, 017112. doi: 10.1088/1752-7155/7/1/017112.
14. Montuschi, P.; Paris, D.; Montella, S.; Melck, D.; Mirra, V.; Santini, G.; Mores, N.; Montemitro, E.; Majo, F.; Lucidi, V.; Bush, A..; Motta, A..; Santamaria, F. Nuclear magnetic resonance-based metabolomics discriminates primary ciliary dyskinesia from cystic fibrosis. *Am. J. Respir. Crit. Care Med.* **2014** *190(2)*, 229–233. doi: 10.1164/rccm.201402-0249LE.

15. Gilchrist, F.J.; Belcher, J.; Jones, A.M.; Smith, D.; Smyth, A.R.; Southern, K.W.; Španěl, P.; Webb, A.K.; Lenney, W. Exhaled breath hydrogen cyanide as a marker of early Pseudomonas aeruginosa infection in children with cystic fibrosis. *ERJ Open Res.* **2015** *1(2)*, 00044–2015. doi: 10.1183/23120541.00044-2015.
16. Antus, B.; Barta, I.; Csiszer, E.; Kelemen, K. Exhaled breath condensate pH in patients with cystic fibrosis. *Inflamm. Res.* **2012** *61(10)*, 1141–1147. doi: 10.1007/s00011-012-0508-9.
17. Smith, D.; Sovová, K.; Dryahina, K.; Doušová, T.; Dřevínek, P.; Španěl, P. Breath concentration of acetic acid vapour is elevated in patients with cystic fibrosis. *J. Breath Res.* **2016** *10(2)*, 021002. doi: 10.1088/1752-7155/10/2/021002.
18. Bos, L.D.; Meinardi, S.; Blake, D.; Whiteson, K. Bacteria in the airways of patients with cystic fibrosis are genetically capable of producing VOCs in breath. *J Breath Res.* **2016** *10(4)*, 047103. doi: 10.1088/1752-7163/10/4/047103.
19. Neerincx, A.H.; Geurts, B.P.; van Loon, J.; Tiemes, V.; Jansen, J.J.; Harren, F.J.; Kluijtmans, L.A.; Merkus, P.J.; Cristescu, S.M.; Buydens, L.M.; Wevers, R.A. Detection of Staphylococcus aureus in cystic fibrosis patients using breath VOC profiles. *J. Breath Res.* **2016** *10(4)*, 046014. doi: 10.1088/1752-7155/10/4/046014.
20. Španěl, P.; Sovová, K.; Dryahina, K.; Doušová, T.; Dřevínek, P.; Smith, D. Do linear logistic model analyses of volatile biomarkers in exhaled breath of cystic fibrosis patients reliably indicate Pseudomonas aeruginosa infection? *J. Breath Res.* **2016** *10(3)*, 036013. doi: 10.1088/1752-7155/10/3/036013.
21. van Mastrigt, E.; Reyes-Reyes, A.; Brand, K.; Bhattacharya, N.; Urbach, H.P.; Stubbs, A.P.; de Jongste, J.C.; Pijnenburg, M.W. Exhaled breath profiling using broadband quantum cascade laser-based spectroscopy in healthy children and children with asthma and cystic fibrosis. *J. Breath Res.* **2016** *10(2)*, 026003. doi: 10.1088/1752-7155/10/2/026003.
22. Pabary, R.; Huang, J.; Kumar, S.; Alton, E.W.; Bush, A.; Hanna, G.B.; Davies, J.C. Does mass spectrometric breath analysis detect Pseudomonas aeruginosa in cystic fibrosis? *Eur. Respir. J.* **2016** *47(3)*, 994–997. doi: 10.1183/13993003.00944-2015.
23. Zang, X.; Monge, M.E.; McCarty, N.A.; Stecenko, A.A.; Fernández, F.M. Feasibility of Early Detection of Cystic Fibrosis Acute Pulmonary Exacerbations by Exhaled Breath Condensate Metabolomics: A Pilot Study. *J. Proteome Res.* **2017 16(2)**, 550–558. doi: 10.1021/acs.jproteome.6b00675.
24. Gaisl, T.; Bregy, L.; Stebler, N.; Gaugg, M.T.; Bruderer, T.; García-Gómez, D.; Moeller, A.; Singer, F.; Schwarz, E.I.; Benden, C.M-L.; Sinues, P.; Zenobi, R.; Kohler, M. Real-time exhaled breath analysis in patients with cystic fibrosis and controls. *J. Breath Res.* **2018** *12(3)*, 036013. doi: 10.1088/1752-7163/aab7fd.
25. Zang, X.; Monge, M.E.; Gaul, D.A.; McCarty, N.A.; Stecenko, A.; Fernández, F.M. Early Detection of Cystic Fibrosis Acute Pulmonary Exacerbations by Exhaled Breath Condensate Metabolomics. *J. Proteome Res.* **2020** *9(1)*, 144–152. doi: 10.1021/acs.jproteome.9b00443.
26. van Horck, M.; Smolinska, A.; Wesseling, G.; de Winter-de Groot, K..; de Vreede, I.; Winkens, B.; Jöbsis, Q.; Dallinga, J.; Dompeling, E.; van Schooten, F.J. Exhaled volatile organic compounds detect pulmonary exacerbations early in children with cystic fibrosis: results of a 1 year observational pilot study. *J. Breath Res.* **2021** *15(2)*, 026012. doi: 10.1088/1752-7163/abda55.
27. Kos, R.; Brinkman, P.; Neerincx, A.H.; Paff, T.; Gerritsen, M.G.; Lammers, A.; Kraneveld, A.D.; Heijerman, H.G.M.; Janssens, H.M.; Davies, J.C.; Majoor, C.J.; Weersink, E.J.; Sterk, P.J.; Haarman, E.G.; Bos, L.D.; Maitland-van der Zee, A.H. Targeted exhaled breath analysis for detection of Pseudomonas aeruginosa in cystic fibrosis patients. *J. Cyst. Fibros*. **2022** *21(1)*, e28–e34. doi: 10.1016/j.jcf.2021.04.015.
28. Woollam, M.; Siegel, A.P.; Grocki, P.; Saunders, J.L.; Sanders, D.B.; Agarwal, M.; Davis, M.D. Preliminary method for profiling volatile organic compounds in breath that correlate with pulmonary function and other clinical traits of subjects diagnosed with cystic fibrosis: a pilot study. *J. Breath Res.* **2022** *16(2)*. doi: 10.1088/1752-7163/ac522f.
29. Mani-Varnosfaderani, A.; Gao, A.; Poch, K.R.; Caceres, S.M.; Nick, J.A.; Hill, J.E. Breath biomarkers associated withnontuberculosis mycobacteriadisease status in persons with cystic fibrosis: a pilot study. *J. Breath Res*. **2022** *16(3)*. doi: 10.1088/1752-7163/ac6bb6.
30. Weber, R.; Perkins, N.; Bruderer, T.; Micic, S.; Moeller, A. Identification of Exhaled Metabolites in Children with Cystic Fibrosis. *Metabolites* **2022** *12(10)*, 980. doi: 10.3390/metabo12100980.
31. Mustafina, M.; Silantyev, A.; Krasovskiy, S.; Chernyak, A.; Naumenko, Z.; Suvorov, A.; Gognieva, D.; Abdullaev, M.; Bektimirova, A.; Bykova, A.; Dergacheva, V.; Betelin, V.; Kopylov, P. Exhaled breath analysis in adult patients with cystic fibrosis by real-time proton mass spectrometry. *Clin. Chim. Acta* **2024** *560*, 119733. doi: 10.1016/j.cca.2024.119733.

32. Veit, G.; Avramescu, R.G.; Chiang, A.N.; Houck, S.A.; Cai, Z.; Peters, K.W. et al. From CFTR biology toward combinatorial pharmacotherapy: expanded classification of cystic fibrosis mutations. *Mol. Biol. Cell* **2016** *27(3)*, 424–433. 15
